# Genome-wide association analysis identifies natural allelic variants associated with panicle architecture variation in African rice, *Oryza glaberrima* Steud

**DOI:** 10.1093/g3journal/jkad174

**Published:** 2023-08-03

**Authors:** Fabrice Ntakirutimana, Christine Tranchant-Dubreuil, Philippe Cubry, Kapeel Chougule, Jianwei Zhang, Rod A Wing, Hélène Adam, Mathias Lorieux, Stefan Jouannic

**Affiliations:** DIADE, University of Montpellier, IRD, CIRAD, 34394 Montpellier, France; DIADE, University of Montpellier, IRD, CIRAD, 34394 Montpellier, France; DIADE, University of Montpellier, IRD, CIRAD, 34394 Montpellier, France; Cold Spring Harbor Laboratory, Cold Spring Harbor, NY 11724, USA; Arizona Genomics Institute, School of Plant Sciences, University of Arizona, Tucson, AZ 85721, USA; National Key Laboratory of Crop Genetic Improvement, Hubei Hongshan Laboratory, Huazhong Agricultural University, Wuhan 430070, China; Arizona Genomics Institute, School of Plant Sciences, University of Arizona, Tucson, AZ 85721, USA; Center for Desert Agriculture, Biological and Environmental Sciences & Engineering Division (BESE), King Abdullah University of Science and Technology (KAUST), Thuwal 23955, Saudi Arabia; DIADE, University of Montpellier, IRD, CIRAD, 34394 Montpellier, France; DIADE, University of Montpellier, IRD, CIRAD, 34394 Montpellier, France; DIADE, University of Montpellier, IRD, CIRAD, 34394 Montpellier, France

**Keywords:** rice, *Oryza glaberrima*, GWAS, panicle architecture, PHYB gene, flowering time, plant genetics and genomics

## Abstract

African rice (*Oryza glaberrima* Steud), a short-day cereal crop closely related to Asian rice (*Oryza sativa* L.), has been cultivated in Sub-Saharan Africa for ∼ 3,000 years. Although less cultivated globally, it is a valuable genetic resource in creating high-yielding cultivars that are better adapted to diverse biotic and abiotic stresses. While inflorescence architecture, a key trait for rice grain yield improvement, has been extensively studied in Asian rice, the morphological and genetic determinants of this complex trait are less understood in African rice. In this study, using a previously developed association panel of 162 *O. glaberrima* accessions and new SNP variants characterized through mapping to a new version of the *O. glaberrima* reference genome, we conducted a genome-wide association study of four major morphological panicle traits. We have found a total of 41 stable genomic regions that are significantly associated with these traits, of which 13 co-localized with previously identified QTLs in *O. sativa* populations and 28 were unique for this association panel. Additionally, we found a genomic region of interest on chromosome 3 that was associated with the number of spikelets and primary and secondary branches. Within this region was localized the *O. sativa* ortholog of the *PHYTOCHROME B* gene (*Oglab_006903/OgPHYB*). Haplotype analysis revealed the occurrence of natural sequence variants at the *OgPHYB* locus associated with panicle architecture variation through modulation of the flowering time phenotype, whereas no equivalent alleles were found in *O. sativa*. The identification in this study of genomic regions specific to *O. glaberrima* indicates panicle-related intra-specific genetic variation in this species, increasing our understanding of the underlying molecular processes governing panicle architecture. Identified candidate genes and major haplotypes may facilitate the breeding of new African rice cultivars with preferred panicle traits.

## Introduction

Panicle architecture is one of the essential morphological traits that directly influence grain yield in rice. Panicle architecture in rice is determined through a combination of different traits, including panicle rachis length, panicle branch number, and spikelet number (Crowell *et al*. 2016)⁠. The improvement of panicle architectural traits has long interested rice farmers and breeders. It has been reported that high-yielding rice cultivars produce longer primary branches and more secondary branches than low-yielding genotypes (Agata *et al*. 2020)⁠. Although an incremental increase in the sink size of the panicle has resulted in considerably more spikelets in modern rice cultivars, several genotypes have failed to reach the expected grain yield potential due to poor grain filling. Therefore, attaining a balance between panicle traits is vital to optimize grain yield and this remains paramount in breeding programs (Panigrahi *et al*. 2019)⁠. Rice panicle architecture is a complex quantitative trait controlled by multiple genes and greatly influenced by environmental signals. By using QTL mapping, a number of genes associated with panicle development were detected, several of which directly affect grain yield, such as *Gn1a* (Ashikari *et al*. 2005)⁠, *DEP1* (Huang *et al*. 2009)⁠, *IPA1/OsSPL14* (Jiao *et al*. 2010; Miura *et al*. 2010)⁠, *qSrn7*/*FZP* (Fujishiro *et al*. 2018)⁠, *Prl5*, and *Pbl6* (Agata *et al*. 2020). Furthermore, several genes influencing panicle architecture variation were identified through mutant characterization. For instance, the *ABERRANT PANICLE ORGANIZATION1 (APO1)* (Ikeda *et al*. 2007)⁠, *LAX PANICLE1 (LAX1)* (Komatsu *et al*. 2001)*⁠*, *FRIZZY PANICLE (FZP)* (Komatsu *et al*. 2003)*⁠*, and *TAWAWA1 (TAW1)* (Yoshida *et al*. 2013)*⁠* genes determine panicle architecture in rice by modulating branching patterns and the number of spikelets. Although the identification of these genes has aided the elucidation of the molecular basis of panicle development in rice, their application in breeding programs is challenging because most of the corresponding mutants produce plants with abnormal morphological traits. Therefore, the natural sequence variations of key panicle-related genes have been studied in several rice populations and several superior haplotypes associated with panicle traits have been identified (Jang *et al*. 2018; Abbai *et al*. 2019)⁠. Although the divergent selection of such superior alleles has aided in diverse rice breeding initiatives (Miura *et al*. 2010; Yan *et al*. 2011; Lin *et al*. 2020)⁠, a large number of alleles potentially associated with panicle traits and grain yield remain to be fully exploited. In this connection, the identification of genes and large-effect alleles linked with panicle architecture phenotype will facilitate future targeted genetic modifications to improve yield and enrich genetic diversity.

Flowering time (FT) or heading date is another important character known to influence rice yield-related traits in relation to a wide range of environmental cues (Huang *et al*. 2012)⁠. Flowering time phenotype was a recurrent target of selection during rice domestication and breeding. The introgression of superior gene alleles related to flowering from wild rice species, several of which are linked with panicle development and yield variations, revealed a great potential for the genetic improvement of cultivated genotypes (Yan *et al*. 2011)⁠. Impressive examples exist for the *GRAIN NUMBER PLANT HEIGHT AND HEADING DATE 7* and *8* (*Ghd7* and *Ghd8)* genes (Gao *et al*. 2014; Lin *et al*. 2020)⁠ for which introgression of superior alleles of these genes from diverse genetic resources has resulted in a late heading phenotype under long-day conditions and a consequent rice grain improvement yield by modulating primary and secondary branches (Xue *et al*. 2008; Lu *et al*. 2012)⁠. Flowering time phenotype in photosensitive rice is determined by photoperiod, the seasonal change in day-length, as one of the important environment signals for plants. Rice as the model for short-day species has been extensively studied to understand the photoperiodic control of its flowering pathway (Fan and Zhang 2018; Andrade *et al*. 2022; Molla 2022)⁠. Two independent regulatory pathways involving the *GIGANTEA*, *HEADING DATE 1* and *HEADING DATE 3a* (*OsGI-Hd1-Hd3a*) rice genes were identified to regulate heading date phenotype in rice under short-day and long-day conditions. The *Hd1* gene activates rice flowering under short-day conditions by up-regulating the *Hd3a* gene (Kim *et al*. 2007)⁠. When rice is in long-day conditions, however, *Hd1* down-regulates *Hd3a* to activate flowering. Moreover, day-length measurement by photoreceptors is essential to ensure flowering success in rice (Song *et al*. 2010)⁠. Phytochrome (PHY) members are the essential red/far-red light receptors of plant species. The rice genome contains three phytochrome genes (*PHYA*, *PHYB*, and *PHYC*), with each gene playing a distinct, but partially redundant, role in light-mediated developmental processes, including floral induction (Kay *et al*. 1989; Takano *et al*. 2005)⁠. Molecular analyses indicated that rice phytochromes are day-length sensitive with the *phyb* mutant exhibiting late flowering under both short- and long-day conditions and the *phyc* mutant repressing flowering only under short-day conditions (Osugi *et al*. 2011)⁠. By contrast, the *phya* mutant exhibits an early flowering phenotype under both short- and long-day signals. It was also shown that rice phytochromes *PHYA* and *PHYB* directly interact with *Ghd7* by opposing *OsGI*-mediated *Ghd7* degradation, thus delaying flowering under long-day conditions (Weng *et al*. 2014)⁠.

African rice (*Oryza glaberrima* Steud.) is a short-day cereal crop closely related to Asian rice (*Oryza sativa* L.) and has been cultivated in west Sub-Saharan Africa for ∼ 3,000 years (Linares 2002; Cubry *et al*. 2018)⁠. Compared with Asian rice, African rice is less grown globally, but it possesses special traits that are valued to improve Asian rice, including strong resistance to diseases, pests, poor and acid soils, and environmental stresses (Wu *et al*. 2017; Choi *et al*. 2019)⁠. With regard to panicle architecture, African rice displays a sparse panicle with fewer spikelets caused by lower number of secondary branches compared with Asian rice, suggesting a divergent genetic control of panicle architecture phenotype between the two species (Ta *et al*. 2017; Harrop *et al*. 2019; Reyes *et al*. 2021)⁠. According to several studies, the wide genetic diversity between Asian rice and African rice could serve as pool of potential genes for varietal improvement between these species (Oladokun 2006; Ishizaki and Kumashiro 2008)⁠. The most well-known example of introgression between the two species is the development of New Rice for Africa (NERICA) cultivars through recurrent back-crosses, which resulted in better resistance to biotic and abiotic stresses compared to Asian rice and an improved grain yield with respect to African rice (Bocco *et al*. 2012; Wang *et al*. 2014)⁠. In addition, researchers working on *O. glaberrima* populations have attempted to dissect the morphological traits and associated genetic mechanisms underlying the domestication of this species. A study by Ta *et al*. (2017)⁠ revealed that *O. glaberrima* has wider inflorescence meristems and more extensive branching patterns, resulting in larger numbers of spikelets compared to its wild progenitor *Oryza barthii.* The authors suggested that this variation is a result of modifications in the expression of genes that act early in the determination of branching patterns.

Given that advances in sequencing technologies have provided useful genomic resources for several rice species, genome-wide association studies (GWAS) have become popular in rice, especially *O. sativa,* and have helped efforts to dissect causal biological mechanisms underlying various agronomically important traits (Song *et al*. 2018; Wang *et al*. 2020)⁠, including those related to panicle architecture (Rebolledo *et al*. 2016; Reig-Valiente *et al*. 2018; Ta *et al*. 2018; Bai *et al*. 2021)⁠. However, only a few GWAS studies have been performed on *O. glaberrima*, some of which were based on traits related to salinity tolerance (Meyer *et al*. 2016)⁠ and transpiration efficiency (Affortit *et al*. 2022)⁠. A recent GWAS study by Cubry *et al*. (2020)⁠, which employed a panel of 163 *O. glaberrima* genotypes (Cubry *et al*. 2018)⁠, identified several QTLs associated with flowering time, resistance to *Rice yellow mottle virus* (RYMV), and panicle morphological traits, providing valuable resources for genetic analyses of agronomic traits in this species. Although several QTLs related to panicle architecture phenotype have been identified, the key genes and major effect alleles underlying panicle-related traits in African rice remain less well documented, and a global view of how genes related to flowering time interact with panicle traits in African rice is still lacking.

Here, we evaluate the genetic mechanisms modulating panicle architecture across 162 *O. glaberrima* accessions obtained from a previously genotyped association panel representing a wide geographical range from West Africa to East Africa (Cubry *et al*. 2018)⁠. For the purpose of this study, we used new SNP/InDel datasets obtained from mapping to an improved reference genome for *O. glaberrima* acc. CG14 (Tranchant-Dubreuil *et al*. 2022)⁠. Genome-wide association scans for primary branch number (PBN), secondary branch number (SBN), spikelet number (SpN), and rachis length (RL) revealed loci linked to each trait and identified genomic regions for candidate gene and major allele identification. We discuss the effects of allelic variation at the *OgPHYB* locus on variations in flowering time and panicle morphological traits. The findings of this study will contribute to the understanding of the genetic basis of panicle morphological traits in the African rice. By dissecting the genetic mechanisms underlying these traits, our research provides crucial insights into the specificities of *O. glaberrima* compared to the Asian species *O. sativa*. These findings can be applied in future rice breeding programs to improve panicle characteristics and enhance the productivity of African rice crops.

## Methods

### Plant material, growing conditions, and measurements

The association panel under study is composed of 162 traditional accessions of *O. glaberrima* which originated mainly from West Africa, with some accessions sampled from Central East Africa. Additional details on this association panel and field experiments were previously described by Cubry *et al*. (2020)⁠⁠. Briefly, the seedlings of all accessions were planted for phenotypic evaluation at the Institut de l’Environnement et de Recherches Agricoles (INERA) station in 2012 and 2014 under irrigated conditions. The field experiment was established using an alpha-lattice design with two replicates. Plants were sown at two different periods in the same year: the first at the beginning of June (“early sowing”) and the second in mid-July (“late sowing”). Plants were grown in 0.5 m^2^ plots with 15 plants per plot. FT was observed for both early and late sowing over 2 years and was recorded as number of days from sowing to the date when 50% of the plants of an accession displayed heading panicles. About 14 days after the heading date, the three main panicles per accession per replicate were collected (i.e. nine panicles/accession/sowing date/replicate) from central plants to measure panicle traits, from the early sowing only, over the 2 years. These panicles were photographed and analyzed using the P-TRAP software (AL-Tam *et al*. 2013)⁠. Eight phenotypic traits, including RL, SpN, PBN, SBN, primary branch length (PBL), secondary branch length (SBL), primary branch internode length (PBintL), and secondary branch internode length (SBintL) were assessed.

### Phenotype statistical analyses

The data for panicle traits used in this study have been previously assessed for normality by Cubry *et al*. (2020)⁠. As all panicle traits exhibited significant deviations from a normal distribution, the Box and Cox transformation method (Box and Cox 1964)⁠ was employed to identify the optimal transformation for each panicle trait, ensuring compliance with statistical model assumptions, including normally distributed error terms and constant variance. The best linear unbiased estimate (BLUE) for each accession was estimated for all the transformed traits using a mixed linear model fitted in the lme4 R package (Zhang *et al*. 2011)⁠. The model incorporated accession as a fixed effect and year, as well as the year *x* accession interaction, as random effects. To calculate the broad-sense heritability (*H^2^*) of each trait, the model was modified by treating all the variables as random effects. The variance component estimates obtained from each fitted model were utilized to estimate the broad-sense heritability following the methodologies described by Oakey *et al*. (2006)⁠. To identify which traits explained the most phenotypic variation among the *O. glaberrima* accessions under study, principal component analysis (PCA) was performed on the yearly phenotypic means and the BLUEs using the dudi.pca() function from ade4 R package (Dray and Dufour 2007)⁠. Pearson's correlation analyses and corresponding probability values were estimated between all pairwise combinations of traits using the chart.Correlation() function implemented in R package PerformanceAnalytics (Peterson *et al*. 2014)⁠. Before conducting BLUE-based correlation analyses, the BLUEs of each trait were subjected to a back-transformation procedure to restore them to their original scale.

### Genotypic data

We used a set of 162 *O. glaberrima* accessions, which were previously subjected to high-depth re-sequencing (Cubry *et al*. 2018)⁠, to generate the SNP/InDel markers used in the present study. Paired-end read was filtered by quality (*q* < 20). Filtered reads were mapped to the CG14 reference genome (Accession GCA_000147395, https://www.ebi.ac.uk/ena/browser/view/GCA_000147395; Tranchant-Dubreuil *et al*. 2022)⁠ using Burrow–Wheeler Aligner software v0.7.4 (Li and Durbin 2009)⁠. Reads mapped in proper pairs were extracted using the SAMtools-view command from SAMtools v1.3.1 (Li *et al*. 2009)⁠. SNP/InDel variants were called for each accession using the Haplotype Caller (emit-ref-confidence GVCF mode) module in GATKv4.1.9.0 (McKenna *et al*. 2010; Auwera *et al*. 2013)⁠. The GATK Genomics DBImport and GenotypeGVCFs modules were employed for joint genotyping to produce raw VCF files for each accession. Raw SNP/InDel variants were filtered using GATK VariantFiltration based on the following criteria: quality higher than 200, depth coverage between 10 and 20,000, and less than three SNPs/InDels within a 10-bp window. The SNPs and InDels identified by GATK were further filtered using BCFtools v1.16 (Danecek *et al*. 2021)⁠ and VCFtools v0.1.16 (Danecek *et al*. 2011)⁠ by applying the following criteria: only biallelic SNPs/InDels; minor allele frequency (MAF) of 5%; maximum missing data of 20%; and homozygous-variant called in more 90% of samples. The remaining missing genotypes were imputed using the impute() function of the LEA R package (Gain and François 2021)⁠. All filtered SNP and InDel variants were annotated according to *O. glaberrima* acc. CG14 (version OglaRS2) genome annotation (https://ftp.gramene.org/oryza/release-6/gff3/oryza_glaberrima/) using SnpEff software v5.1 (Cingolani *et al*. 2012)⁠. Genes were functionally annotated by aligning their protein sequences against the NCBI's non-redundant database using the BLASTP v.2.12.0+ (e-value cut-off of 10^−6^) (Camacho *et al*. 2009)⁠. InterPro protein domain searches were performed using the software InterProScan v5.53–87 (Quevillon *et al*. 2005)⁠ based on these parameters: -appl pfam -dp -goterms -iprlookup -pa. GO annotations were characterized with the tool Blast2GO v6.0.3 (Conesa *et al*. 2005)⁠ using default parameters.

### Linkage disequilibrium and population structure analysis

Linkage disequilibrium (LD) between SNPs in the 162 accessions was estimated using the squared correlation coefficient (*r^2^*) using the PopLDdecay software (Zhang *et al*. 2019)⁠. LD data were summarized by estimating the mean LD between a pair of SNPs in 1,000 bp bins and was plotted against physical distance with a LOESS curve fitted to visualize LD decay. The population structure of the 162 accessions was evaluated using sparse nonnegative matrix factorization (snmf) function in the LEA R package (Gain and François 2021)⁠ which implements the admixture model (Chikhi *et al*. 2001)⁠ to estimate ancestry proportions. The cross-entropy criterion was employed to calculate varying levels of ancestral groups (*K* = 1–10) and 10 replications were used for each *K*. To further assess the population genetic structure of our panel, PCA was carried out using the -pca command in PLINK version 1.9 (Chang *et al*. 2015)⁠.

### Genome-wide association analysis

We conducted GWAS to identify genomic regions associated with panicle traits. SNP-trait association analyses were performed using 687,436 high-quality SNP markers while correcting for population structure. The association analyses focused on four panicle traits (SpN, PBN, SBN, and RL) with high phenotypic variation and known to contribute to panicle branching biological processes, specifically the number and order of branching. To capture the overall trait structure and variability, GWAS was also conducted using the first three principal components (PC1 to PC3) derived from the BLUEs of all eight panicle traits analyzed in this study. The GWAS was performed for each of these traits as described by Cubry *et al*. (2020)⁠.⁠ Two models were considered in this study: (1) the latent factor mixed model (LFMM) implemented in LFMM v2 R package (Caye *et al*. 2019)⁠; (2) Fixed and random model Circulating Probability Unification (FarmCPU) implemented in the GAPIT R package (Lipka *et al*. 2012)⁠. LFMM tests for association of the phenotypes with each SNP marker were conducted with adjustment for confounding of population structure and other hidden variables by regression on the four latent factors. For FarmCPU, which implements a multi-locus linear mixed model and iteratively a fixed effect model and a random effect model (REM) to avoid model overfitting (Liu *et al*. 2016)⁠, we employed four PCs to control spurious associations. FarmCPU incorporates the kinship matrix (K) estimated from associated markers as an additional covariate. GWAS analyses were initially conducted separately for each year using phenotypic means, and *P*-values obtained for the same phenotypic trait were subsequently combined across the 2 years using Stouffer's method (Riley, 1949)⁠. Furthermore, to account for the potential influence of genotype × year variance, additional GWAS analyses were performed using the BLUEs of the four panicle traits. FDR estimation was performed for each trait to account for multiple testing, but the FDR-adjusted *P*-values were overly stringent, resulting in a very limited number of significant SNP-trait associations. Hence, a 10^−4^*P*-value threshold was employed to select candidate SNP-trait associations. Only association peaks identified under both LFMM and FarmCPU methods were considered for further analyses. Manhattan plots for all the associations and their corresponding quantile-quantile (QQ) plots were drawn using the qmplot Python package (Huang and Libiseller-Egger 2022)⁠⁠. Candidate SNP-trait associations were characterized in silico for the identification of genomic regions and search for potential candidate genes. To define genomic regions for the selection of candidate genes, local LD decay was employed and was calculated within a 1,000 kb region upstream and downstream of the significant SNP markers. LD heatmaps surrounding the significant GWAS peaks were constructed using the LDBlockShow software using *r^2^* > 0.6 (Dong *et al*. 2021)⁠. Local Manhattan plots and genomic structure lollipop plots were generated using KaryoploteR package (Gel and Serra 2017)⁠. Association peaks identified under both LFMM and FarmCPU methods were considered for the screening of putative candidate genes, which was based on the functional annotation of the *O. glaberrima* acc. CG14 reference genome (version OglaRS2).

### Haplotype analysis

Haplotype analysis was performed for genomic regions and candidate genes using the Pegas R package (Paradis and Barrett 2010)⁠. Haplotypes within genomic regions were inferred using SNPs that met the *P* < 10^−4^ threshold in the GWAS analysis, focusing on regions exhibiting multiple candidate SNP-trait associations in the LD block. Only haplotypes represented by at least five accessions (∼ 3% of the entire population) were considered for further analyses. Two main haplotypes (high- and low-value haplotypes) were identified for each genomic region based on significantly differential phenotypic values. The polymorphic sites including both SNPs and InDels shared by two major haplotypes across all the accessions were assessed. The phenotypic values of different haplotypes were compared using a Welsh *t*-test (*P* ≤ 0.05), allowing unequal variances between haplotypes. The haplotypes of identified candidate genes were defined using all the SNPs and InDels present within the gene after removing the variants with MAF < 0.05 and missing data > 0.2.

## Results

### Variation of *O. glaberrima* panicle traits

To understand the phenotypic variation of panicle architecture in African rice, we used an association panel of 162 genotypes grown in 2012 and 2014. Eight traits related to panicle architecture, including PBN, SBN, SpN, RL, PBL, SBL, PBintL, and SBintL, were assessed. Information relating to the mean, range, standard deviation, and coefficient of variation (CV) of the traits measured can be found in Supplementary Table 1. Overall, a wide range of phenotypic variability was observed among the 162 *O. glaberrima* accessions across 2 years. All the traits, except for RL, PBL, and PBintL, showed a CV higher than 20%. The highest phenotypic variation was observed for SBN (CV = 48.41%) followed by SpN (CV = 27.07%), SBintL (CV = 25.45%), and PBN (CV = 20.97). The panicle traits, namely SpN, PBN, PBintL, RL, and SBL, demonstrated moderate to relatively high broad-sense heritability scores ranging from 0.53 to 0.66 (Supplementary Table 1), highlighting a substantial contribution of genetic factors to the observed variation in these traits. Conversely, traits such as PBL, SBN, and SBintL exhibited lower broad-sense heritability scores (0.33–0.41), indicating a more pronounced influence of environmental factors on the phenotypic variability of these traits.

**Fig. 1.**
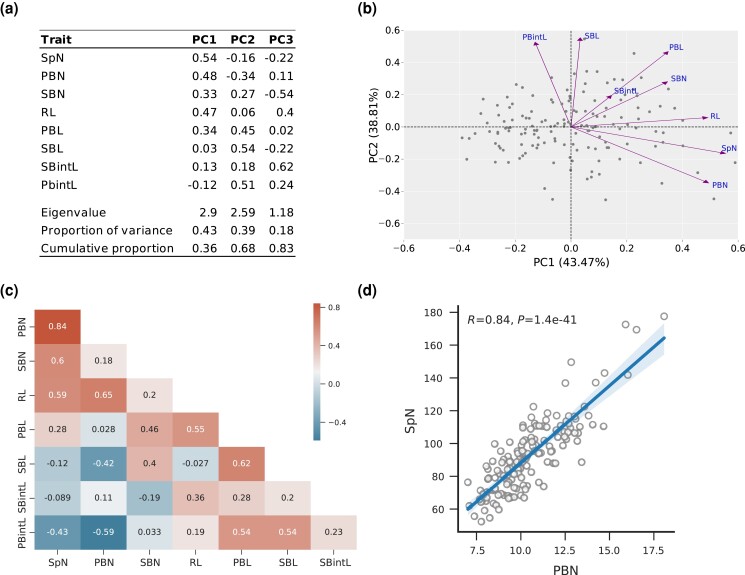
Phenotypic analysis revealing panicle traits relationships. a) PCA of panicle traits using BLUE values and summary of the first three principal components (PC1, PC2, and PC3) for the eight panicle traits analyzed in *O. glaberrima* population. b) Loading plot of PC1 and PC2, illustrating the loadings of each panicle trait on these principal components. The percentages of variance explained by PC1 and PC2 are provided in parentheses. c) A heatmap depicting Pearson's correlation coefficients among the BLUE values of panicle traits across all accessions of the panel. d) Scatter plot showing phenotypic correlation of PBN and SpN in the full panel.

To explore the relationships among the panicle traits and identify underlying factors contributing to trait variation, PCA analyses were conducted using yearly phenotypic means and the BLUEs for all eight panicle traits. When considering the BLUEs, PC1 explained 43.47% of the total variance (Fig. 1, a and b). Notably, traits such as SpN, PBN, and RL exhibited positive loadings greater than 0.4 on PC1 (0.47–0.54), indicating that individuals with higher PC1 scores possessed larger panicle sizes, increased primary branches, and a higher number of spikelets per panicle. PC2 explained 38.81% of the total variance and exhibited high loadings for traits such as SBL, PBL, and PBintL (0.45–0.54). This indicates that plants with higher PC2 scores had longer secondary branch length, primary branch length, and panicle internode length. Furthermore, the SBN trait displayed a high negative score on PC3 (−0.54), suggesting that plants with higher PC3 scores had a lower number of secondary branches. The results of the PCA using both the BLUEs and yearly phenotypic means showed consistent patterns, with slight variation observed for the 2012 phenotypic means (Supplementary Fig. 1, a and b). These findings suggest that PC1 and PC2 can serve as quantitative indices for characterizing panicle architecture. To explore the phenotypic relationships among the eight studied traits, Pearson's correlation coefficients were calculated using both the BLUEs (Fig. 1c) and yearly phenotypic means (Supplementary Fig. 1, a and b). The correlation patterns observed were consistent between the BLUEs and phenotypic means. The SpN trait showed a significant positive correlation with PBN (*R* = 0.84, Fig. 1, c and d). This correlation persisted across both years, with a particularly strong association observed in 2014 (Supplementary Fig. 1c). Similarly, SpN showed a significant positive correlation with SBN and RL with Pearson's correlation coefficients of 0.59 and 0.60, respectively (Fig. 1c). PBN exhibited a significant positive correlation with RL (*R* = 0.65). Overall, traits related to length, such as SBL, PBintL, and SBintL, showed negative relationships with traits related to number, such as SpN, PBN (Fig. 1, b and c, Supplementary Fig. 1, a and b).

### Variant identification, population structure, and linkage disequilibrium

In the present study, a set of 162 *O. glaberrima* genotypes that were previously fully sequenced (Cubry *et al*. 2018)⁠ were used for variant identification. After raw read filtering, alignment to the CG14 OglaRS2 reference genome, variant calling, and initial filtering, we obtained a total of 6,851,320 SNP/InDels, with an average of 25 variants per kb. After discarding variants with MAF < 5%, missing data > 20%, and proportion of heterozygous-variants > 10%, we reduced this number to 687,436 high-quality SNPs and ∼ 400 K high-quality InDels.

**Fig. 2.**
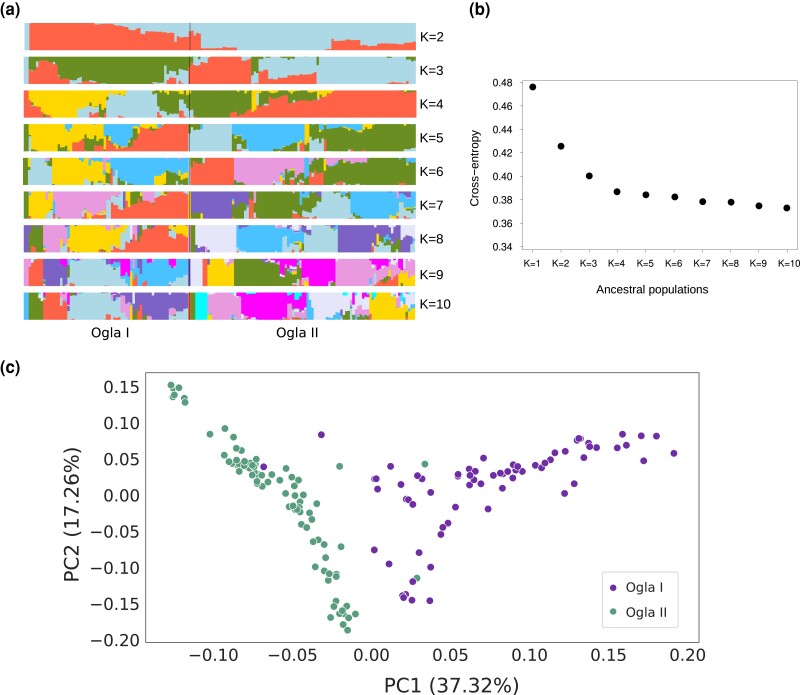
Population structure analysis of 162 *O. glaberrima* accessions. a) Population structure of the panel inferred using snmf() function from LEA R package. The 162 accessions were assigned into two groups (Ogla I and Ogla II). Each color denotes one population. b) Cross-entropy criterion shows the number of populations that best explains the panel under study. Maximum likelihood was observed at *K* = 4, which indicates the four populations from the panel under study. c) PCA of 162 *O. glaberrima* accessions based on all SNP markers. PC1 and PC2 denote the score of principal components 1 and 2, respectively. The proportion of variance explained by PC1 and PC2 is indicated in parentheses.

Using SNP markers, we assessed the population structure of the current panel using sparse nonnegative matrix factorization (snmf), which employs a Bayesian model-based method for clustering. Ancestral populations were identified by varying levels of K means from 2 to 10 groups (Fig. 2a). With *K* = 2, the accessions of the panel were divided into two clusters corresponding to the Ogla I and Ogla II groups identified by Orjuela *et al*. (2014)⁠. Increasing *K* levels divided these two groups into admixtured subgroups. A cross-entropy criterion indicated a plateau at *K* = 4, assuming optimum number of ancestral groups at this point (Fig. 2b). Hence, for the purpose of this study, *K* = 4 was retained for subsequent association analysis. A PCA was performed based on SNP markers to evaluate the consistency of the ancestral groups identified by admixture analysis. The PCA results indicated the first two PCs accounted for the Ogla I and Ogla II groups, with PC1 and PC2 explaining 37.32% and 17.26% of total genetic variance, respectively (Fig. 2c). Taken together, our population structure and PCA analyses indicated that the genotypes of this association panel do not depict a strongly structured population.

**Fig. 3.**
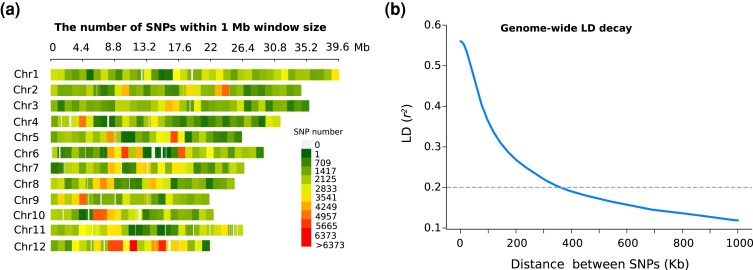
SNP variants and LD decay of 162 *O. glaberrima* genotypes. a) SNP distribution and density along the 12 chromosomes of *O. glaberrima* accessions. The colors correspond to the number of SNPs in a 1-Mb region. b) Genome-wide average LD decay indicated by relationship of smoothed (*r^2^*) values and physical distance between SNP pairs. The horizontal dashed line, along the *x*-axis corresponding to physical positions, depicts the LD threshold of 0.2 for pairwise *r^2^*.

Within our association panel, the average genome-wide LD decays to *r^2^* = 0.2 at ∼ 350 kb (Fig. 3), which is comparable with a previous study in *O. glaberrima* (Meyer *et al*. 2016)⁠. The magnitude of LD decay varied considerably among different chromosomes (Supplementary Fig. 2). The LD decay rate for all chromosomes except chromosomes 6, and 10 decayed to *r^2^* = 0.2 at a distance between SNP markers varying from ∼ 92 kb (Chr2) to ∼ 600 kb (Chr3). Strong patterns of LD were detected for chromosomes 6 and 10, with *r^2^* = 0.2 for up to 1,000 kb. The very high extent of LD observed for some chromosomes could result in the inclusion of a large number of candidate genes within LD blocks which can complicate the search of candidate genes from significant peaks. Thus, we employed local LD decay (*r^2^* = 0.6) around significant peaks to define genomic regions for candidate gene disclosure.

### Detection of genomic regions associated with panicle traits by GWAS

**Fig. 4.**
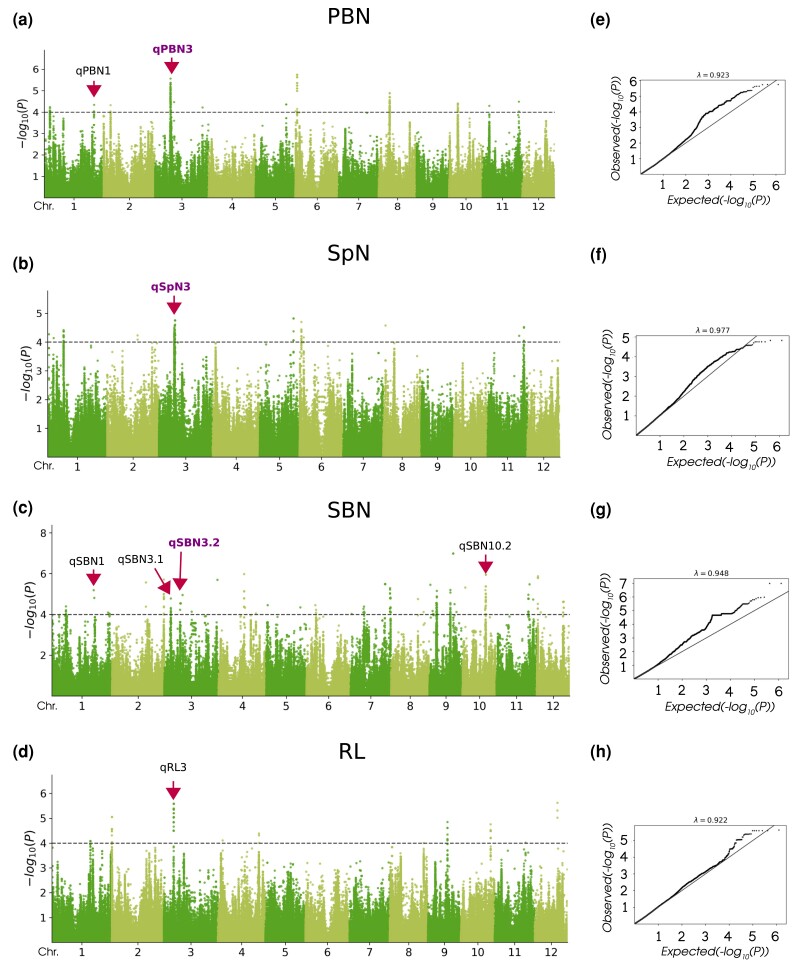
Genome-wide association mapping using LFMM method. (a–d) Manhattan plots using two years of phenotypic data for PBN, SpN, SBN, and RL. The dashed black lines represent the genome-wide significance threshold (−log_10_*P* = 4). (e–h) QQ plots for the panicle traits tested. Arrows correspond to the candidate regions that co-localized with genes associated with panicle traits. The candidate regions in purple denote genomic regions on chromosome 3, which contain overlapping associations.

To identify associations between SNP markers and variations in the panicle traits under study in a collection of 162 *O. glaberrima* accessions, we employed the set of 687,436 high-quality SNPs (MAF > 0.05 with no missing data) uniformly distributed across the 12 *O. glaberrima* chromosomes. The phenotypic values of the evaluated traits were previously checked for normality and were transformed (Cubry *et al*. 2020)⁠. In our GWAS analyses, BLUEs were utilized instead of best linear unbiased predictions to address potential issues with double shrinking. Comparing the GWAS results obtained with BLUEs and the yearly phenotypic means showed a concordance, particularly in the main regions (Fig. 4, Supplementary Figs. 3–5). However, the use of BLUEs led to the exclusion of some GWAS regions featuring a single SNP-trait association at 10^−4^*P*-value threshold, predominantly observed with the FarmCPU method (Supplementary Figs. 3 and 4). GWAS analyses using panicle traits identified 1,351 associations (*P*-value < 10^−4^) between 1,302 SNPs and 1,140 associations (*P*-value < 10^−4^) between 1,132 SNP markers across all 12 chromosomes for LFMM (Fig. 4, a–d) and FarmCPU (Supplementary Fig. 5, a–d) methods, respectively. The QQ plots indicated that the two GWAS methods fitted well to the data, the observed *P*-values being uniformly distributed with some apparent inflation producing higher values compared to the expected *P*-values (Fig. 4, e–h, Supplementary Fig. 5, e–h). Using PC scores, we detected a total of 746 and 326 associations (*P*-value < 10^−4^) for LFMM and FarmCPU, respectively, with 210 associations overlapping between the two methods (Supplementary Fig. 6, a–f).

We focused on those association signals that were detected using both the LFMM and FarmCPU methods to reduce the proportion of spurious associations. Based on the PC scores, a total of 14 GWAS sites were identified, with five regions specific to PC1, six regions specific to PC2, and two regions shared by both PC1 and PC2 (Supplementary Fig. 6, a–f). However, only two regions were associated with PC3, providing further support for our hypothesis that PC1 and PC2 effectively capture the variation in panicle architecture within the studied *O. glaberrima* population. GWAS results of the four panicle traits identified a total of 41 candidate regions that were common to the two methods, with 29 supported by more than one SNP-trait association at 10^−4^*P*-value threshold (Table 1, Supplementary Table 2). The largest number of candidate regions was observed for the SBN trait with a total of 23 regions distributed throughout all chromosomes except chromosomes 6 and 8. The SpN and RL traits were associated with 9 and 11 genomic regions, respectively. The smallest number of genomic regions (6) was observed for the PBN trait. Notably, we detected several genomic regions that contained significant associations for more than one trait. A genomic region on chromosome 6, which overlapped with associations for PC1, exhibited associations with both SpN and PBN. Additionally, two regions on chromosome 11, which overlapped with associations for PC1 and PC2, demonstrated associations with both SpN and SBN traits. These findings suggest that these genomic regions play a crucial role in regulating the key panicle traits contributing to the variation captured by PC1 and PC2 in *O. glaberrima*.

**Fig. 5.**
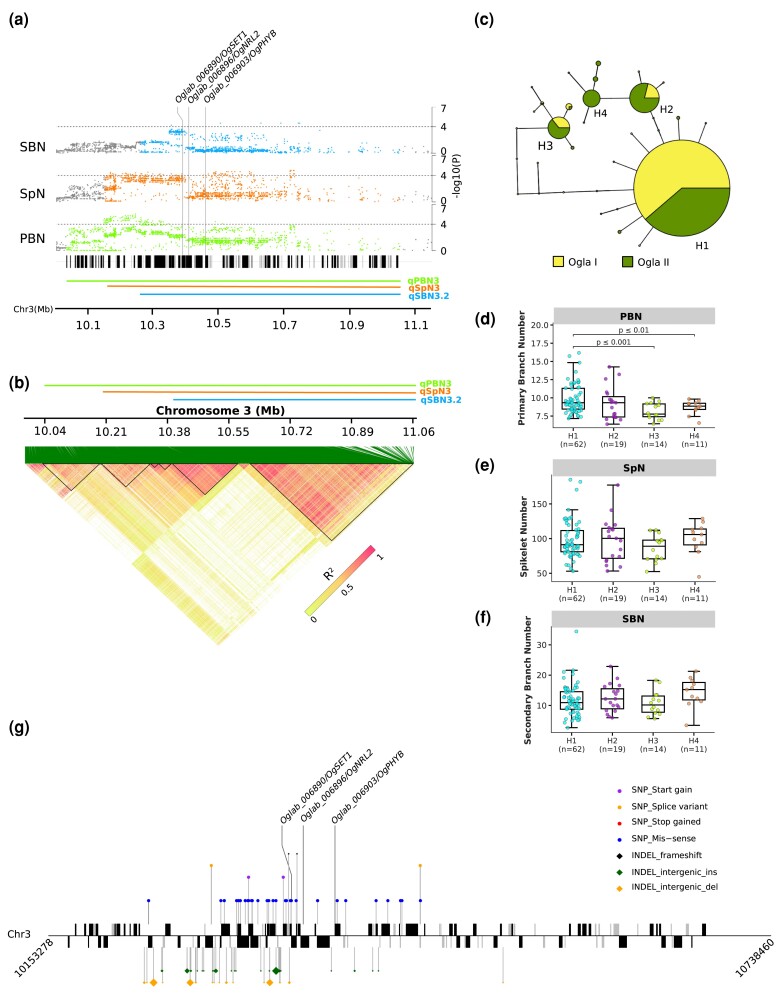
Genomic organization and haplotype analysis for the overlapping regions on chromosome 3. a) Local Manhattan plots for qPBN3 (bottom), qSpN3 (middle), and qSBN3.2 (top). Green, red, and blue lines indicate the physical position of qPBN3, qSpN3, and qSBN3.2 respectively. The *x*-axis represents the physical location of SNPs across each genomic region under study, and the *y*-axis represents −log_10_*P*-values. The ideogram represents the genes that are expressed (black) and not expressed (gray) in the panicle based on the publicly available databases and RNA-seq data. Candidate genes known to be associated with yield potential are highlighted. b) Heatmap showing LD patterns for the chromosomal region around qPBN3, qSpN3, and qSBN3.2. Green, red, and blue horizontal lines indicate the physical position, and black triangles are the observed LD blocks for qPBN3, qSpN3, and qSBN3.2, respectively. LD is depicted by *r^2^* statistic. The light lime to red gradient depicts the range of *r^2^* values. c) Haplotype network analysis using significant SNP markers observed in the region. Haplotypes are denoted by circles with size corresponding to the number of accessions carrying that haplotype. (d–f) Boxplots with individual dots for PBN, SpN, and SBN based on four major haplotypes (*n* > 5 accessions), namely H1, H2, H3, and H4. The frequency for each haplotype is highlighted below the *x*-axis label. The statistical difference between haplotypes was assessed by Welch's *t*-test. g) Lollipop plot showing the polymorphisms shared among accessions carrying haplotypes H1 and H3. The schematic view of the candidate region (middle) depicts genes expressed in the panicle (black) and genes not expressed in the panicle (gray) according to the publicly available databases and RNA-seq data. The genes that are on the forward strand (positive strand) are presented above the *x*-axis and those that are on the reverse strand (negative strand) are presented below the *x*-axis. The different polymorphic sites between H1 and H3 are represented by colored lollipops. To simplify the plot, only SNPs/InDels affecting protein sequences were represented (excluding synonymous changes and UTR/intronic sites) as well as only the INDELs in the intergenic regions (full SNP/InDel list in Supplementary Table 4).

Unlike the correlations we observed between RL and number-related traits, such as SpN and PBN, no common genomic region was shared between RL and these traits, suggesting that the genetic network controlling RL operates independently of other panicle traits tested in this study. On chromosome 3, we found a genomic region for PBN which overlapped with genomic regions for SpN and SBN traits (Fig. 4, a–c). Notably, this candidate region also overlapped with associations for PC1, as determined by the LFMM method (Supplementary Fig. 6a), providing evidence for the genetic underpinnings of their co-variation observed in PC1. Based on local Manhattan plots and LD, the candidate region was delineated to 10.039–10.053 Mb based on pairwise LD correlation (*r^2^* > 0.6) and consists of three genomic regions (Fig. 5a): qPBN3 (positions 10,038,624 to 11,052,717), qSpN3 (positions 10,157,280 to 11,052,717), and qSBN3.2 (positions 10,266,019 to 11,052,717). The genomic regions qPBN3 and qSpN3 consisted of 114 and 85 SNPs, respectively, that exceeded the threshold of *P* < 10^−4^ in the GWAS analyses using the LFMM method, of which 30 were common to both regions (Table 1, Supplementary Table 2). The qSBN3.2 region contained six SNPs that exceeded the threshold of *P* < 10^−4^ based on the LFMM method and never shared exactly the same significant SNPs with qPBN3 and qSpN3.

**Table 1.** GWAS regions associated with panicle architecture-related traits obtained using LFMM and FarmCPU methods.

GWAS site	Trait	Chr.	Physical position		Significant SNPs		Colocated genes (OglaRS2)
			Start	End	LFMM	FarmCPU	
qPBN1	PBN	1	33,064,198	34,064,198	3	5	*Oglab_004326/OgqSH1*
							*Oglab_004365/OgQHB*
qSpN1.1	SpN	1	861,855	872,749	1	1	
qSpN1.2	SpN	1	10,605,869	10,650,025	38	24	
qSBN1	SBN	1	27,511,040	27,989,047	2	2	*Oglab_003422/OgMADS32*
qPBN2	PBN	2	4,616,401	4,650,455	2	21	
qSBN2.1	SBN	2	22,294,110	22,327,093	1	1	
qRL2	RL	2	329	124,799	12	13	
qSBN2.2	SBN	2	34,066,865	34,144,989	13	6	
qSBN3.1	SBN	3	4,238,189	4,262,556	9	1	*Oglab_005985/OgMADS47*
qRL3	RL	3	6,397,785	6,501,994	40	41	*Oglab_006320/OgBRR1*
qPBN3	PBN	3	10,038,624	11,052,717	114	39	*Oglab_006903/OgPHYB*
							*Oglab_006890/OgSET1*
							*Oglab_006896/OgNRL2*
qSpN3	SpN	3	10,157,280	11,052,717	85	5	
qSBN3.2	SBN	3	10,266,019	11,052,717	6	5	
qSBN3.3	SBN	3	11,980,837	11,991,756	1	1	
qSBN4.1	SBN	4	16,885,547	17,048,779	8	4	
qSBN4.2	SBN	4	17,560,791	17,618,502	1	1	
qRL4	RL	4	27,321,847	27,322,998	3	1	
qSBN5	SBN	5	22,448,666	22,458,772	1	1	
qSpN5	SpN	5	22,467,801	22,491,369	6	3	
qPBN_SpN6	PBN&SpN	6	1,428,722	1,443,690	22	28	
qSBN7.1	SBN	7	7,764,781	10,028,291	6	261	
qSBN7.2	SBN	7	22,721,981	22,772,209	7	7	
qSBN7.3	SBN	7	25,959,373	26,319,903	23	11	
qSpN8	SpN	8	17,725,053	17,867,343	1	1	
qSBN9.1	SBN	9	612,106	711,298	1	1	
qSBN9.2	SBN	9	4,548,529	4,956,162	36	7	
qSBN9.3	SBN	9	13,780,682	13,827,435	15	8	
qSBN9.4	SBN	9	15,684,086	15,698,634	2	2	
qSBN9.5	SBN	9	17,802,099	17,884,077	1	1	
qRL9	RL	9	129,49,371	12,958,859	8	5	
qPBN10	PBN	10	5,683,746	5,935,141	27	61	
qSBN10.1	SBN	10	1,553,226	1,922,495	1	1	
qSBN10.2	SBN	10	15,071,623	15,418,109	236	226	*Oglab_036752/OgLAC19*
qRL10	RL	10	19,710,668	19,725,628	13	13	
qRL11	RL	11	20,212,990	20,549,402	1	1	
qSpN_SBN11.1	SpN&SBN	11	20,936,988	21,091,576	121	93	
qSpN_SBN11.2	SpN&SBN	11	24176983	24192991	3	1	
qPBN_SpN11	SpN&PBN	11	24,240,766	24,244,301	2	1	
qSBN12.1	SBN	12	800,654	1,066,600	6	6	
qRL12	RL	12	15,089,822	15,114,283	3	3	
qSBN12.1	SBN	12	17,527,841	17,922,786	8	6	

As some panicle morphological traits tested in this study exhibited a phenotypic relationship with flowering time (Supplementary Fig. 7), we performed a GWAS analysis for flowering time to test whether significant panicle-related traits associations from this region could be confounded with associations to flowering time. GWAS results identified significant association signals on chromosome 3 (positions 10,038,624 to 12,730,361) associated with flowering time assessed for early sowing (DFTa) based on the LFMM method (Supplementary Fig. 8, a and b), some of which overlapped with some GWAS peaks for the SpN and PBN traits. However, these association signals were not detected using the FarmCPU method (Supplementary Fig. 8, c and d).

We also assessed whether the GWAS signals detected in this study overlapped with known QTL sites detected in other GWAS studies and mapping populations relating to panicle morphology. To find co-locations of genomic regions of this study with QTL sites previously reported in *O. sativa*, we first converted the coordinates of the GWAS regions of this study into corresponding *O. sativa* coordinates using NUCmer alignment (Marçais *et al*. 2018)⁠. The results revealed a total of 36 QTLs across all 12 chromosomes from previously reported QTLs which co-localized with 13 candidate regions identified in the present study (Supplementary Table 2). Only two of these co-localized QTLs shared the same traits with our GWAS regions, namely qPBN-10 and Q_127 on chromosome 10, which co-localized with qPBN10 for the PBN trait and qRL10 for the RL trait, respectively. Other co-localized QTL sites had been previously mapped for different panicle traits and/or grain yield-related traits. In addition, we compared the GWAS regions found in this study with those detected by Cubry *et al*. (2020)⁠ for panicle traits, the latter study having employed the same association panel but an *O. sativa* reference genome for SNP calling and different criteria for defining candidate regions. Nine overlaps were identified, three of which shared the same traits, namely two genomic regions for the RL trait localized on chromosomes 3 and 9 and one genomic region for the SBN trait located on chromosome 12 (Supplementary Table 2).

### Identification of candidate genes and haplotypes associated with panicle-related traits

Several genes ascribed to rice panicle development have been characterized and some have been cloned (Li, Cheng, *et al*. 2021)⁠. To assess whether any of these genes could be linked with trait-associated markers, we evaluated the regions around the peaks delineated by haplotype blocks with strong LD patterns (*r^2^* > 0.6). Our study identified several candidate genes that mapped within or flanking GWAS regions displaying a relationship to panicle development and architecture. Moreover, by using significant markers, we identified haplotypes for the 28 genomic regions that encompass at least two significant SNP markers. The two main haplotypes were classified into high-value and low-value haplotypes based on their effects on panicle traits tested here. These analyses were focused on genomic regions that co-localized candidate genes related to panicle architecture, although consistent results were obtained for other regions (Supplementary Table 3). Subsequently, the polymorphisms shared by two major haplotypes were evaluated to elucidate functional polymorphic sites.

With respect to the GWAS locus on chromosome 3 containing three overlapping candidate regions (qPBN3, qSpN3, and qSBN3.2), the region in question co-localized with three candidate genes associated with the development of the panicle (Table 1; Fig. 5, a and b). A cluster of highly significant SNPs associated with PBN and SpN traits along with a significant SBN signal was located in the sixth block near the *Oglab_006890/OgSET1* gene orthologous to *O. sativa SET PROTEIN 1* (*OsSET1*) (Fig. 5, a and b). This gene encodes an enhancer of zeste [E(Z)] homolog, a key component of the Polycomb Repressive Complex2 (PRC2), that is involved in short day signaling to mediate the accurate photoperiodic control of flowering time (Liang 2003; Lu *et al*. 2013; Liu *et al.* 2014)⁠. Another cluster of significant signals for PBN, SpN, and SBN traits located in the seventh block overlapped with a gene encoding the ortholog of *O. sativa PHYTOCHROME B* gene (*Oglab_006903/OgPHYB*). The rice *PHYB* gene was shown to be involved in day-length-dependent flowering time regulation (Ishikawa *et al*. 2011; Gao *et al*. 2014)⁠. Overexpression of this gene in rice results in pleiotropic effects including reduced panicle number and total grain yield (Hu *et al*. 2020)⁠. Another candidate gene, *Oglab_006896/OgNRL2*, encoding an ortholog of *NARROW AND ROLLED LEAF 2* (*NRL2*) gene (Zhao *et al*. 2016)⁠, was mapped at 21.24 kb from the marker in block six (position 10,390,700) that showed significant association with the SpN trait, and 35.87 kb from the marker in block seven (position 10,456,656) that also showed significant association with SpN trait, but was in weak LD with its closest flanking markers. The *NRL2* gene has pleiotropic effects on vegetative organs, reproductive organs, male sterility, and grain shape (Zhao *et al*. 2016; Xu *et al*. 2018)⁠. Furthermore, we employed all the significant SNP markers from this region to evaluate haplotype diversity. The results showed 32 distinct haplotypes (Fig. 5c), with four haplotypes shared by at least five accessions. Of these haplotypes, two main ones (H1 and H3) exhibited significant differences for PBN values (Fig. 5d). When looking at SpN and SBN however, genotypes harboring H1 exhibited a slight increase in phenotypic values for these traits compared to those carrying H3, although the differences were not significant (NS) (Fig. 5, e and f). We evaluated the polymorphisms shared by genotypes carrying haplotypes H1 and H3. The results indicated that the region contained 691 polymorphisms shared among accessions carrying H1 and H3, including both SNPs and InDels (Supplementary Table 4). Of these polymorphisms, 40 were localized in the coding regions of 24 genes, including *OgPHYB* and *OgSET1*, and 72, including 4 InDels, were localized in the intergenic regions (Fig. 5g, Supplementary Table 4).

On chromosome 3, we identified another peak (qRL3) encompassing 40 significant SNPs associated with the RL trait. The candidate region was mapped from 6.40 Mb to 6.50 Mb based on pairwise LD correlations (*r^2^* > 0.6), and contained seven LD blocks (Supplementary Fig. 9, a and b). In the third block, a significant SNP (position 6,455,795; −log_10_(*P*) = 4.84) was mapped in the coding region of the *Oglab_006320/OgBRR1* gene orthologous to *BLAST RESISTANT RELATED 1* (*OsBRR1*), which encodes a leucine-rich repeat receptor-like kinase (Supplementary Fig. 9) involved in several developmental and defense-related activities (Peng *et al*. 2009)⁠. This gene is also closely related to the *A. thaliana BARELY ANY MERISTEM 1* and *2* genes (*BAM1* and *BAM2*) that play an important role in meristem activities and in the development of male organs (DeYoung *et al*. 2006; Wang *et al*. 2022)⁠. Another GWAS signal found in the seventh block (position 6,491,017) was mapped in the intergenic region at 7.9 kb from the *Oglab_006323/OgGS3.1* gene orthologous to *O. sativa GRAIN SIZE3.1* (*GS3.1*) encoding a MATE (multidrug and toxic compounds extrusion) transporter, regulating grain size and flavonoid and lignin biosynthesis (Zhang *et al*. 2021)⁠. When conducting haplotype analysis using 40 significant SNPs present in this region, eight haplotypes were found, with two main haplotypes (*n* > 5 accessions), designated as H1 and H2 (Supplementary Fig. 9c, Supplementary Table 5). Comparative phenotype analysis revealed that accessions carrying H1 have globally higher RL values (*P* ≤ 0.01) than those carrying the H2 haplotype (Supplementary Fig. 9d). The analysis identified 42 polymorphisms shared by these two main haplotypes, including a non-synonymous variant (position 6,455,795) within the *Oglab_006320/OsBRR1* gene. No polymorphisms were shared by the two haplotypes observed for the *OgGS3.1* gene.

On chromosome 10, a genomic region for SBN (qSBN10.2) was mapped from 15.072 Mb to 15.418 Mb (346.49 kb) with a cluster of signals with strong LD values (Supplementary Fig. 10). This region contained 236 significant markers based on the LFMM method (Table 1, Supplementary Table 2), 14 of which mapped in the coding region of the *Oglab_036752/OgLAC19* gene, which encodes the ortholog of the *O. sativa LACCASE 19* (*OsLAC19*) gene. The *O. sativa OsLAC19* regulates lignin biosynthesis involved in plant development and stress responses in rice (Liu *et al*. 2017)⁠. *LACCASE* genes were also reported to play an important role in panicle elongation when overexpressed in rice (Swetha *et al*. 2018)⁠. Haplotype analysis using significant SNPs of this QTL identified 19 haplotypes (Supplementary Fig. 6c, Supplementary Table 6), of which two main ones (H1 and H2) showed distinct values for the SBN (Supplementary Fig. 10d). Accessions carrying H1 showed significantly higher SBN values compared to those carrying H2. Next, we assessed the polymorphic sites shared by these two major haplotypes and found 275 variants, including an InDel variant (position 15,345,029) leading to a translation stop gain and 11 non-synonymous variants observed for the *Oglab_036752/OsLAC19* gene (Supplementary Fig. 10).

In addition to these candidate genes, we noted that some strong GWAS signals detected in this study were found within or in close proximity to genes whose functions in panicle development are not yet known. This is the case for the most significant SNP for the PBN trait localized in the qPBN6_SpN6 region on chromosome 6 (position 1,441,882; −log_10_(*P*) = 5.74), which was mapped in the coding region of *Oglab_018111* orthologous to the *O. sativa LOC_Os06g03390* gene. The *LOC_Os06g03390* gene was annotated as a homolog to a *NUCLEOTIDE BINDING SITE–LEUCINE-RICH REPEAT* (*NBS-LRR*) gene (Wang *et al*. 2013)⁠, but its potential function in panicle development remains to be demonstrated. Moreover, the SNP showing the strongest association with the SpN trait in this study located in the qSpN5 region (position 22,482,789; −log_10_(*P*) = 4.82) on chromosome 5 mapped closely to various hypothetical genes, none of which is known to be related to panicle architecture.

### Association of the *OgPHYB* gene to panicle morphological traits and flowering time variation

**Fig. 6.**
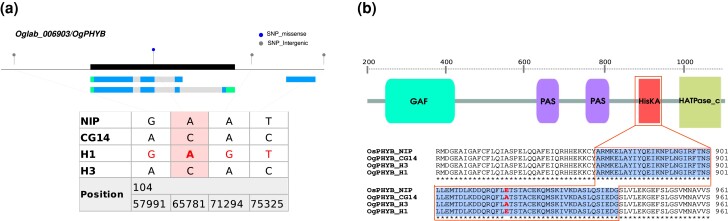
Polymorphism of *OgPHYB* gene between H1 and H3 haplotypes. a) A lollipop plot depicting variants observed in the region containing 5 kb upstream and downstream of *OgPHYB* gene between haplotypes H1 and H3. The black box represents *OgPHYB* gene structure. The green, gray, and blue boxes below the *x*-axis represent UTRs, introns, and CDS regions of the two annotated transcripts, respectively. b) Full protein structure and amino acid sequence alignment of *OgPHYB* and *OsPHYB* proteins in the region of HisKA domain for the *O. glaberrima* H1 and H3 haplotypes and *O. sativa* cv. Nipponbare. The different protein domains are indicated by colored boxes.

To further characterize the nucleotide diversity in the GWAS locus on chromosome 3 where the qPBN3, qSpN3, and qSBN3.2 regions were co-localized, we analyzed in more detail the polymorphic sites within this region. Amongst the multiple polymorphisms identified, we detected two frameshift InDels (positions 10,388,879 and 10,402,042) leading to high impact at protein level for two annotated genes of unknown function (Supplementary Table 4). In addition, 34 non-synonymous SNPs were observed in the coding region of 22 genes. We focused on the *OgSET1, OgNRL2,* and *OgPHYB* genes for further analysis, considering polymorphisms from haplotypes H1 and H3 that were located within and around (5 kb upstream and downstream) these genes. Regarding *OgNRL2*, no H1/H3 polymorphic sites were identified in the vicinity of this gene, suggesting that it is not of significance within this genomic region in relation to panicle architecture. The *OgSET1* gene was characterized by two non-synonymous SNPs (positions 10,400,716 and 10,400,736) shared by haplotypes H1 and H3. Concerning the *OgPHYB* gene, only a C/A mutation located in exon-2 (position 10,465,781) of this gene was detected in the two haplotypes, with H3 accessions carrying the reference allele C (i.e. that of the CG14 accession) and H1 accessions carrying the alternate A allele (Fig. 6a). Sequence comparison between the two haplotypes at the *OgPHYB* locus revealed that this mutation led to a non-synonymous amino acid change of A–E at position 917 in the protein sequence of H1 accessions, and that the mutation is located in the HisKA protein domain (Fig. 6b) known to be involved in signal transduction. Further sequence comparisons indicated that the *O. sativa* OsPHYB protein from the reference genome *O. sativa* ssp. *japonica* cv Nipponbare carries the E amino acid (C allele in nucleic sequence), as for *O. glaberrima* haplotype H1 (Fig. 6, a and b).

Given that the analysis of *OgPHYB* and *OgSET1* gene polymorphic sites suggested functionally distinct alleles in this association panel, we investigated in more detail the haplotype structure of these genes based on SNPs and InDels detected for the whole *O. glaberrima* population. A total of 11 SNPs/InDels were detected for the *OgSET1* gene in the full *O. glaberrima* panel, with three variants leading to non-synonymous changes at protein level. Haplotype analysis identified eight haplotypes, with three main haplotypes (i.e. representing more than five accessions) covering 97% of the full population (Supplementary Tables 3 and 7). Haplotype and phenotype association analysis revealed that the three main haplotypes (Ha, Hb, and Hc) did not exhibit a significant difference for SpN and PBN traits (Supplementary Fig. 11). Significant differences in the SBN trait were observed for these three haplotypes, with higher values detected for accessions harboring Hc than for those harboring Ha and Hb.

**Fig. 7.**
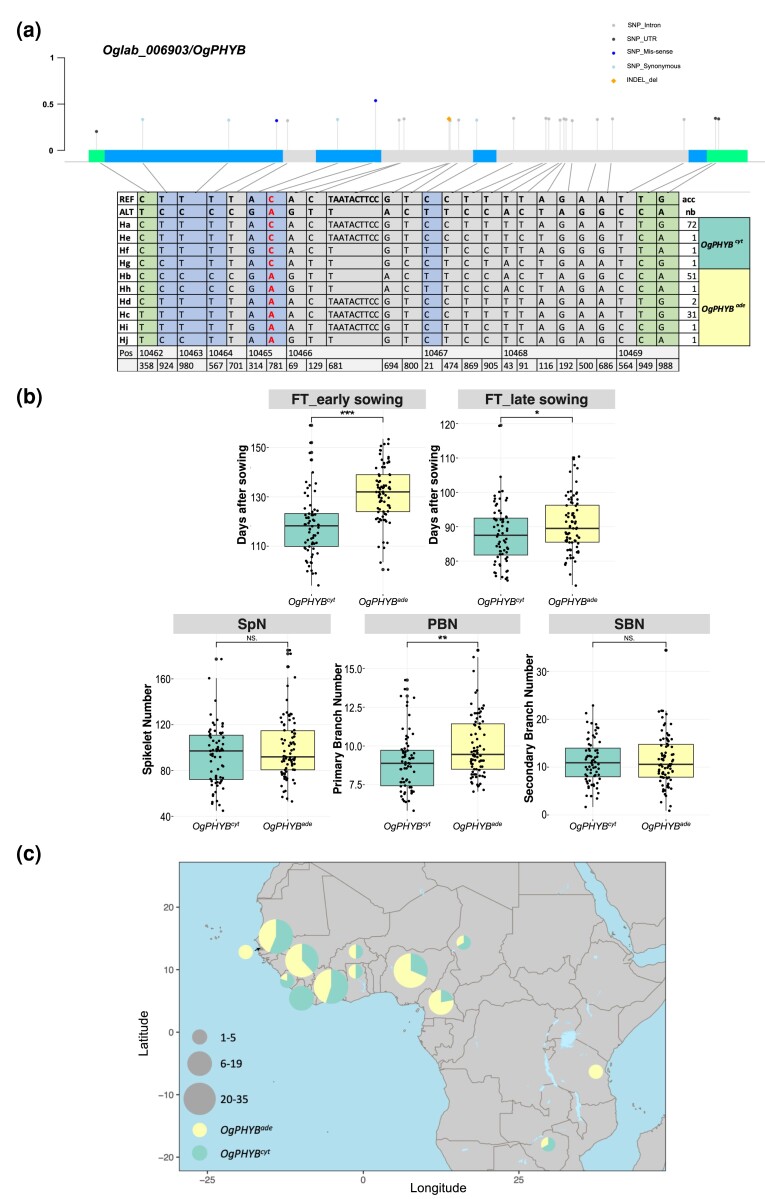
Allelic diversity and geographical distribution of *OgPHYB* in the whole *O. glaberrima* panel. a) Haplotype analysis of *OgPHYB* (bottom) in 162 *O. glaberrima* genotypes using all the SNPs/InDels within the gene. Two haplogroups were formed based on a synonymous variant (position 10465781). *OgPHYB^cyt^* accessions carry cytosine (C) allele, while *OgPHYB^ade^* accessions carry adenine (A) allele. The gene structure and polymorphic sites and their frequency in *O. glaberrima* population are shown at the top. The different polymorphic sites within the gene are represented by colored lollipops. The green, gray, and blue boxes represent UTRs, introns, and CDS regions of the longest *OgPHYB* annotated transcript, respectively. b) Boxplots with individual dots for flowering time assessed at early and late sowing, PBN, SpN, and SBN based on two haplogroups *OgPHYB^cyt^* and *OgPHYB^ade^*. The statistical differences between haplogroups were statistically assessed using Welch's *t*-test (NS; *P < 0.05, **P < 0.01, and ***P < 0.001). c) Geographical distribution of *OgPHYB^cyt^* and *OgPHYB^ade^* haplogroups by country in Africa. The proportions of alleles observed in a given country are denoted by pie charts with size corresponding to the number of accessions carrying that allele.

A total of 25 SNPs/InDels were detected for the *OgPHYB* gene in the full panel, with six sites located in the coding sequence, two of which led to non-synonymous changes at protein level (Fig. 7a, Supplementary Tables 3 and 7). Ten haplotypes were identified in the full panel, among which three (Ha, Hb, and Hc) were carried by more than five accessions (Fig. 7a). The haplotype Ha showed the highest frequency with 72 accessions, while haplotypes Hb and Hc occurred in 51 and 31 accessions, respectively. These 10 haplotypes were classified into two haplogroups based on the non-synonymous SNP in exon 2 of *OgPHYB* (position 10,465,781), namely *OgPHYB^cyt^* for haplotypes with a C allele (e.g. Hb and Hc) and *OgPHYB^ade^* for haplotypes with A allele (e.g. Ha). These haplogroups correspond to the two aforementioned haplotypes H1 and H3 from the overlapping candidate regions qPBN3, qSpN3, and qSBN3.2, with H1 accessions carrying *OgPHYB^ade^* allele and H3 accessions carrying *OgPHYB^ade^* allele. The haplotypes Ha and Hc were distinguished by the C/A mutation at position 10,465,781. The haplotypes Hb and Hc carry both the A allele at position 10,465,781 but possess different alleles in the other five polymorphic sites of the coding sequence. Analyses indicated that 54% of the accessions carry the *OgPHYB^cyt^* allele, while 46% of the accessions carry the *OgPHYB^ade^* allele, indicating that this mutation is present at a high frequency in this *O. glaberrima* panel.

Given the function of the *PHYB* gene and the co-location of the GWAS site for flowering time, we tested the association of *OgPHYB* haplotypes with flowering time and observed that the *OgPHYB^cyt^* alleles were associated with earlier flowering compared to the *OgPHYB^ade^* alleles in both early and late sowing conditions (Fig. 7b). In parallel, the *OgPHYB^ade^* alleles exhibited higher PBN values than the *OgPHYB^cyt^* alleles, while the impact of these alleles on variations of SpN and SBN trait values was not significant. These results illustrate the parallel effect of *OgPHYB* alleles on panicle architecture, especially primary branching, and flowering time. We then evaluated the geographical distribution of the *OgPHYB* haplogroups in Africa (Fig. 7c). The accessions of this panel are distributed mainly in West Africa, with a few accessions from Central East Africa. It was found that most of the 13 countries represented in our *O. glaberrima* panel harbor the two haplogroups, but in different proportions. Although the geographical distribution of these two haplogroups is not clearly distinct, a higher allelic proportion of *OgPHYB^cyt^* appears to be observed in the western part of Sub-Saharan Africa.

An examination of *OsPHYB* haplotypes in the 3 K Rice Genome population (Wang *et al*. 2018)⁠ considering only exonic polymorphic sites (*n* > 5 accessions; MAF > 5%; missing data < 20%) revealed six main haplotypes, of which none showed evidence for polymorphism at the site corresponding to the *OgPHYB^cyt^* and *OgPHYB^ade^* alleles (Supplementary Table 8). This would suggest that this mutation is specific to *O. glaberrima* (or African rice species) or was filtered out in the 3 K Rice Genome datasets.

## Discussion

### Differential contribution of panicle morphological traits to variation in spikelet number between Asian and African rice species

We evaluated the phenotypic data of eight traits related to panicle morphology in a diversity panel of African rice (*O. glaberrima*) over a two-year period. High variability and heritability were observed for four major traits (PBN, SBN, SpN, and RL), with strong correlations detected among them, except for the SBN and RL traits, for which the pairwise relationship was not significant. Compared to the other traits measured here, the PBN and SpN traits contributed most to panicle diversity in this panel. Variation of the spikelet number per panicle in *O. glaberrima* relies most on variation of primary branch number per panicle. As indicated in this study, these traits are more under genetic than environmental influence in this panel. Similar phenotypic relationships were reported in various diversity panels for the Asian rice *O. sativa* (Rebolledo *et al*. 2016; Ta *et al*. 2018; Bai *et al*. 2021)⁠ and bi-parental populations (Li, Zhang, *et al*. 2021)⁠. However, in most of these studies, although not all, the SBN trait contributed more to variation of the SpN than did the PBN trait. This variability in panicle architecture was also reported among *O. sativa* subspecies, with larger panicles observed for the japonica subspecies than for the Indica one. This divergence in the contribution of individual morphological traits to differences in spikelet number per panicle between the two subspecies mirrors morphological differences observed between different rice species. Indeed, the *O. glaberrima* panicle is globally less branched with a lower complexity (i.e. fewer secondary branches) compared to that of *O. sativa* (Harrop *et al*. 2019)⁠, indicating a *de facto* lower contribution of the SBN trait to spikelet number variation in *O. glaberrima* compared to *O. sativa*. This in turn suggests that primary and secondary branching influence spikelet number differently in *O. glaberrima* and *O. sativa*, implying that a divergence in the underlying regulatory mechanisms might exist between the two species.

### Contribution of genomic regions to panicle architectural diversity in *O. glaberrima*

Given the environmental dependency of several panicle traits and extensive LD patterns in rice that are attributable to self-pollination (Crowell *et al*. 2016)⁠, GWAS studies in this species have revealed only a limited number of panicle trait associations. Using the four main morphological components of panicle architecture, namely RL, PBN, SBN, and SpN, we identified a total of 41 genomic regions that are consistent between two GWAS methods, 29 of which were supported by more than one significant SNP. Of these 29 regions, five were associated with two or three morphological traits (e.g. qPBN3/qSpN3/qSBN3.2, qPBN_SpN6, qSpN_SBN11.1, qSpN_SBN11.2, and qPBN_SpN11). These results reflect the relationship between these morphological traits, with branch number contributing to the diversity of spikelet number per panicle. Overall, no overlap was observed between the associations identified for PBN and SBN traits, with the exception of the GWAS signals on chromosome 3 (i.e. qPBN3 and qSBN3.2), for which no significant SNP common to these traits was detected for these traits. This finding would suggest that primary and secondary branch numbers in *O. glaberrima* are controlled by different genetic mechanisms, as similarly reported for *O. sativa* populations (Ta *et al*. 2018; Bai *et al.* 2021)⁠. This is consistent with the panicle development process, in which all the axillary meristems produced on the rachis axis contribute to primary branches, in contrast to the axillary meristems from a primary branch which balance between branch meristem (i.e. leading to a secondary branch) and spikelet meristem (i.e. leading to a lateral spikelet) fates (Itoh *et al*. 2005)⁠. Consequently, the intra-specific diversity for spikelet number per panicle in *O. glaberrima* is, at least in part, related to the rate of axillary meristem establishment from the rachis meristem. Moreover, although the RL trait exhibited a significant phenotypic relationship with the PBN and SBN traits, distinct genomic regions were detected for the RL trait, suggesting that the genetic network controlling this trait operates independently of other panicle traits tested in this study. Differences in genetic determinants between number-related traits and length-related traits have been also reported in several *O. sativa* association panels (Huang *et al*. 2012; Crowell *et al*. 2016; Ta *et al*. 2016)⁠.⁠

Comparison of GWAS results obtained in this study with those from Cubry *et al*. (2020)⁠⁠ performed using the same association panel identified nine overlaps, with only three co-locations detected for similar traits. Compared with the present results, no genomic regions for the SpN and PBN traits, the two main contributors of panicle architecture diversity, were identified in the previous study. The relatively small overlap between the two studies is mostly explained by the different thresholds used to select the significant SNPs (10^−5^*P*-value cutoff in the previous study and 10^−4^ in this study), and to a lesser extent by the different SNP datasets and methodology used to delineate genomic regions. The present study benefits from SNP/InDel datasets obtained through mapping to the high-quality *O. glaberrima* acc. CG14 (version OglaRS2) reference genome (Tranchant-Dubreuil *et al*. 2022), which limited mapping biases from intra-specific divergence. Moreover, this reference genome and a new gene annotation provided here allowed the accurate prioritization of genes from GWAS loci in this study and might be helpful for the dissection of the regulatory mechanisms underlying agronomically important traits in this species.

By comparing the GWAS loci obtained in this study with previously reported QTLs related to panicle morphology derived from GWAS studies and mapping populations in *O. sativa*, 13 GWAS loci were found to co-localize with 37 previously identified QTLs in *O. sativa* populations. Eight QTLs related to the four morphological panicle traits tested in this study were identified, with only two QTLs sharing a similar trait (Yonemaru *et al*. 2010; Crowell *et al*. 2016)⁠. This may correspond to the specificities of the panels used, to the adopted methodologies, or to environmental conditions. In addition, up to 20 co-locations with QTLs from mapping populations were found to be related to other panicle-related traits, such as panicle length, rachis thickness, exertion length, and internode number (Ogawa *et al*. 2021)⁠. This observation is not surprising because related traits might be controlled by linked genetic mechanisms. In addition, several overlaps with QTLs detected in GWAS studies and bi-parental linkage analyses were found to be associated with yield-related traits, such as grain number per panicle, thousand-grain yield, and yield per plant (Yonemaru *et al*. 2012; Zhong *et al*. 2021), mirroring the relationship between panicle architecture and parameters of grain yield in rice. Of note, GWAS associations for the SBN trait on chromosome 4 (e.g. qSBN4.2) co-localized with a cluster of GWAS signals related to panicle and yield traits reported in *O. sativa* (Crowell *et al*. 2016)⁠, suggesting a genomic region of interest.

Taken together, although some common QTLs for panicle-related traits were observed between this panel and diverse *O. sativa* populations and some orthologs of genes identified in *O. sativa* were found to be involved in the control of these traits in *O. glaberrima*, these findings suggest that intra-specific variation in African rice species for panicle architecture might rely more on species-specific factors. In addition, quantitative variations in panicle morphology may be attributable to divergences at different stages of panicle development, at the cultivar, subpopulation or species level, involving differences in the expression of numerous genes (Ikeda *et al*. 2004)⁠.

### *OgPHYB* may play important role in determination of the panicle architecture diversity in *O. glaberrima*

One genomic region of interest corresponds to the overlap of three identified genomic regions on chromosome 3, namely qPBN3, qSpN3, and qSBN3.2, which are associated with the three main traits contributing to panicle architecture diversity. Based on the polymorphic sites present within the diversity panel, we hypothesized that *Oglab_006903/OgPHYB* is a strong candidate gene for this genomic region because: (i) this gene was mapped in an LD block with several association peaks for the PBN, SBN, and SpN traits; (ii) several significant SNPs of this region were found in close proximity to significant SNPs associated with flowering time; and (iii) when comparing the polymorphisms shared by the two major haplotypes (H1 and H3) of this genomic region, one mutation located in exon-2 of *OgPHYB* (position 10,465,781) was found to alter the protein sequence in the HisKA domain involved in signal transduction. GWAS peaks associated with heading date phenotype were previously detected in several *O. sativa* populations. *OsPHYB* was found to be localized near a GWAS signal for photoperiod sensitivity in an *O. sativa* population (Jadamba *et al*. 2022). *OsPHYB* was also co-localized in a QTL for heading date detected in a RIL population resulting from a cross between the japonica variety SN265 and indica variety R99 (Li *et al*. 2018)⁠. These results collectively indicate that *PHYB* contributes to the variation of flowering-time phenotype in diverse genetic backgrounds.

Phytochromes, as the sole photoreceptors for perceiving red/far-red light in rice, are required for critical day-length recognition in relation to flowering time (Takano *et al*. 2005; Ishikawa *et al*. 2011)⁠. The flowering time regulation pathway is well documented in Asian rice *O. sativa,* and numerous additional key genes, including *OsGI* and *Ghd7*, were identified to be involved in photoperiodic flowering pathway controlling the two florigen genes *Hd3a* and *RICE FLOWERING LOCUS T 1 (RFT1)* through the *Hd1* flowering gene (Sun *et al*. 2022; Osnato 2023)⁠. *Hd1* alone can essentially act as a flowering promoter and repressor under short-day and long-day conditions, respectively, through the regulation of *Hd3a*. However, phytochromes are required for the critical day-length recognition (Ishikawa *et al*. 2011)⁠. Previous genetic analyses revealed that when *PHYB* is functional, *Hd1* physically interacts with *Ghd7* to repress the expression of *Hd3a*, thus delaying flowering under short-day conditions (Ishikawa *et al*. 2011)⁠. Under long-day conditions however, two active regulatory pathways were identified: (i) *OsGI* promotes *Ghd7* protein degradation, thus promoting early flowering phenotypes; and (ii) phytochromes, mainly *PHYA* and *PHYB*, compete with the *OsGI-Ghd7* complex to stabilize the *Ghd7* protein, thus delaying flowering (Zheng *et al*. 2019)⁠.

The reported polymorphic site in *OgPHYB* is located in the HisKA domain in the C-terminal output module (OPM) of phytochrome. The OPM mediates dimerization and signal transmission to the downstream effectors, through differential interactions with other proteins necessary for nuclear localization and interactions with several nuclear proteins, including transcription factors (Cheng *et al*. 2021). The *OgPHYB^cyt^* and *OgPHYB^ade^* alleles are associated with differential impact on flowering time with shorter flowering time for *OgPHYB^cyt^* in both long-day and short-day conditions but with higher impact under long-day conditions (June, early sowing). The *OgPHYB^ade^* allele is equivalent to the *OsPHYB* gene in *O. sativa* cv. Nipponbare, while *OgPHYB^cy^*^t^ allele showed no equivalent in the 3 K *O. sativa* genomes panel. *OgPHYB^cyt^*-induced early flowering could mirror the loss of function mutant of *PHYB* in *O. sativa* (Takano *et al*. 2005, 2009). This mutation could impact upon the dimerization, the kinase activity and/or interaction with other protein partners, leading to lower repression of flowering notably through the *Ghd7* gene. Besides the regulation of flowering time, rice phytochromes are key regulators that control a series of events during photomorphogenesis, including de-etiolation and plant shape formation (Takano *et al*. 2005, 2009). Recently, it was shown that rice *PHYA* and *PHYB* genes may synergistically affect anther development and pollen viability (Sun *et al*. 2017). In parallel, it was reported that phytochromes promote vegetative branching in *Sorghum bicolor* and *A. thaliana* through the regulation of *TEOSINTE BRANCHED1* (*TB1*)-like genes by suppressing auxin signaling (Kebrom *et al*. 2006, 2009; Krishna Reddy and Finlayson 2014)⁠, indicating that *PHYB* could have impact on meristem functioning. However, even if *PHYB* transcripts are detected in panicles at early stages of development, until recently there was no evidence that this gene could be directly involved in the panicle branching process through the regulation of meristem activity. Therefore, it would appear more likely that there is an indirect effect of *PHYB* on panicle branching through its role in flowering time regulation.

Panicle number and size (branch number and length) are plastic traits prone to mutual compensation under competition for pre-floral assimilate resources in *O. sativa* (Dingkuhn *et al*. 2015; Adriani *et al*. 2016). A longer vegetative stage would impact upon assimilating resources which in turn would affect reproductive meristem activity at the flowering stage, leading to higher branched panicle in *O. glaberrima*. Cui *et al*. (2019)⁠ have reported that early flowering mutants of rice, including *phyb* mutant, exhibit lower biomass and grain yield per plant. However, the sources of yield loss were different between mutants, with the single *phyb* mutant exhibiting a decrease in the number of grains per panicle and the setting rate. This is supported by genetic studies that indicated that introgressed beneficial alleles of *Ghd7* and *Ghd8* from diverse genetic resources might have interacted to induce a late heading phenotype in rice under long-day conditions, which in turn improved rice grain yield by modulating primary and secondary branches (Xue *et al*. 2008; Lu *et al*. 2012). A similar example was observed for West African pearl millet, in which a polymorphism of another phytochrome-encoding gene, *PgPHYC*, was associated with flowering time diversity, spike length, and adaptation to various ecosystems in West Africa (Saïdou *et al*. 2014; Diack *et al*. 2017; Faye *et al*. 2022)⁠, supporting the strong relationship between flowering time, local adaptation, and yield in crop species in West Africa.

### Conclusions

In this study, a detailed phenotypic analysis of panicle architecture in a diversity panel of *O. glaberrima* revealed that variation in primary branch number per panicle is the main contributor to spikelet number variation. Several GWAS loci related to panicle architecture diversity were identified in *O. glaberrima*, notably a genomic region of interest on chromosome 3 with three overlapping regions affecting numbers of spikelets and primary and secondary branches. We hypothesize that *OgPHYB* is the strongest candidate gene for this genomic region, supporting the concept of a functional relationship between flowering time and panicle architecture, which could reflect adaptation to local ecosystems. Nevertheless, single gene variations do not in general explain fully the genetic basis of rice cultivar adaptation to different ecological regions. Thus, it will be of interest to analyze the geographical distribution of haplotype combinations of panicle and flowering time-related genes. Moreover, most of the genomic regions identified were specific to *O. glaberrima*, rather than being co-localized with the QTLs reported in the Asian rice *O. sativa*, suggesting that intra-specific variation in the African rice species for panicle architecture might rely on specific factors and that *O. glaberrima* may harbor a unique diversity. To apply the findings of this study for rice crop improvement, it is crucial to conduct further characterization and validation of functionally significant polymorphisms. This will not only deepen our understanding of the genetic elements that underlie the diversity and local adaptation of panicle architecture, but also contribute to enhancing yield potential. By harnessing this knowledge, future rice breeding programs can strategically target and improve specific panicle traits, leading to overall improvements in crop productivity in this species. Additionally, the observed phenotypic relationships between panicle traits and flowering time highlight the potential for further investigations using a multi-trait GWAS model. Such an approach would provide a comprehensive analysis of the genetic associations between these traits and flowering time, revealing the intricate relationships between them.

## Supplementary Material

jkad174_Supplementary_Data

## Data Availability

The genotypic data used for association analyses have been deposited in the Zenodo repository at https://doi.org/10.5281/zenodo.7755851. The gff file with structural and functional annotation using *O. glaberrima* reference genome (version OglaRS2) has been deposited at https://ftp.gramene.org/oryza/release-6/gff3/oryza_glaberrima/. The *O. glaberrima* reference genome (version OglaRS2) has been deposited in the ENA repository at https://www.ebi.ac.uk/ena/browser/view/GCA_000147395. All other data are included in this article and/or supporting information. Previously published phenotypic data were used for this work (https://github.com/Africrop/gwas_african_rice). Supplemental material available at G3 online.
