# Supplementary material for: Genome-wide association analysis identifies natural allelic variants associated with panicle architecture variation in African rice, Oryza glaberrima Steud
Source: G3 (Bethesda). 2023 Aug 3;13(10):jkad174. doi: 10.1093/g3journal/jkad174 (PMC10542218; doi:10.1093/g3journal/jkad174)
Supplement: jkad174_Supplementary_Data [file jkad174_supplementary_data.zip › Supplemental_Material_Legends_G3-2023-404399.docx]

**Supporting Information legends**

**Supplementary Table S1**. **Phenotypic variation of morphological panicle traits and flowering time for the *O. glaberrima* panel measured in 2012 and 2014.** Broad-sense heritability (*H^2^*), Range, mean, standard deviation (SD), coefficient of variation (CV), minimum (Min), and maximum (Max). Rachis length (RL), spikelet number (SpN), primary branch number (PBN), secondary branch number (SBN), primary branch length average (PBL), secondary branch length average (SBL), PB internode length average (PBintL), SB internode length average (SBintL), flowering time at early sowing (DFTa), and flowering time at late sowing (DFTb).

**Supplementary Table S2**. **Details about genomic regions consistent in both LFMM and farmCPU methods.** Sig_SNPs: number of significant SNPs using LFMM or FarmCPU methods.

**Supplementary Table S3. Haplotype analysis for 29 candidate regions supported by more than one significant SNP and for *OgPHYB* and *OgSET1* genes in qPBN3**. Haplotype analysis for the 29 candidate regions is based on significant SNPs. Haplotype analysis for *OgPHYB* and *OgSET1* genes in qPBN3 is based on whole SNP/InDel datasets. The phenotypic values and observed haplotypes are provided for each accession. Rare haplotypes in the candidate regions correspond to the haplotypes that appeared in less than 5 accessions.

**Supplementary Table S4. Annotation and impact of SNPs and InDels shared among accessions carrying haplotypes H1 and H3 in qPBN3**.

**Supplementary Table S5.** **Annotation and impact of SNPs and InDels shared among accessions carrying haplotypes H1 and H2 in qRL3.**

**Supplementary Table S6**. **Annotation and impact of SNPs and InDels shared among accessions carrying haplotypes H1 and H2 in qSBN10.2.**

**Supplementary Table S7. Annotation and impact of SNPs and InDels shared among accessions of the *O. glaberrima* diversity panel for *OgPHYB* and *OgSET1* genes.**

**Supplementary Table S8. Haplotype analysis of *OsPHYB* gene in the *O. sativa* 3K genome panel.** Haplotype analysis was carried out using the MBK database (http://www.mbkbase.org/rice) based on the following filtering criteria: haplotypes present in at least five accessions; MAF > 5%; and missing data < 20%. The positions in coding sequence are highlighted in blue, and the positions in UTRs are in green.

**Supplementary Figure S1**. **Principal component analysis (PCA) and Pearson's correlation coefficients between phenotype means measured in 2012 (A) and 2014 (B) for panicle traits across all accessions in the panel**. Contribution of the traits to diversity is indicated by colored scaling. Rachis length (RL), spikelet number (SpN), primary branch number (PBN), secondary branch number (SBN), primary branch length (PBL), secondary branch length (SBL), PB internode length (PBintL), and SB internode length (SBintL). Asterisks indicate significant correlations using a two-tailed t-test (*p < 0.05, **p < 0.01, and ***p < 0.001). (C) Scatter plot showing phenotypic correlation of primary branch number (PBN) and spikelet number (SpN) in the full panel. Blue and orange indicate phenotypic data from 2012 and 2014, respectively.

**Supplementary Figure S2. Overall chromosome-wide decay of linkage disequilibrium (LD).** The y-axis indicates smoothed *r^2^* values. The horizontal dashed lines, along the x-axis corresponding to physical positions, depict the LD thresholds of 0.1 and 0.2 for pair-wise *r^2^*.

**Supplementary Figure S3. Genome-wide association mapping for BLUE values using LFMM method.** Manhattan plots (**A-D**) were drawn using BLUE values for primary branch number (PBN), spikelet number (SpN), secondary branch number (SBN), and rachis length (RL). The dashed black lines represent the genome wide significance threshold (-log_10_ *p* = 4). (**E-H**) Quantile-quantile (QQ) plots for the panicle traits tested.

**Supplementary Figure S4. Genome-wide association mapping for BLUE values using FarmCPU method.** Manhattan plots (**A-D**) were drawn using BLUE values for primary branch number (PBN), spikelet number (SpN), secondary branch number (SBN), and rachis length (RL). The dashed black lines represent the genome wide significance threshold (-log_10_ *p* = 4). (**E-H**) Quantile-quantile (QQ) plots for the panicle traits tested.

**Supplementary Figure S5. Genome-wide association mapping using FarmCPU method.** Manhattan plots (**A-D**) were drawn using two years phenotypic data for primary branch number (PBN), spikelet number (SpN), secondary branch number (SBN), and rachis length (RL). The dashed black lines represent the genome wide significance threshold (-log_10_ *p* = 4). (**E-H**) Quantile-quantile (QQ) plots for the panicle traits tested. Arrows correspond to the candidate regions that co-localized genes associated with panicle traits. The candidate regions in purple denote regions on chromosome 3, which contain overlapping associations.

**Supplementary Figure S6. Genome-wide association mapping using principal components (PCs).** Manhattan plots (**A-D**) were drawn using principal components scores for primary branch number (PBN), spikelet number (SpN), secondary branch number (SBN), and rachis length (RL). The dashed black lines represent the genome wide significance threshold (-log_10_ *p* = 4). (**E-H**) Quantile-quantile (QQ) plots for the panicle traits tested.

**Supplementary Figure S7. Correlation plot for morphological panicle traits and flowering time.** A heatmap representing Pearson’s correlation coefficients between phenotype means of all accessions of the panel observed in 2014. Asterisks indicate significant correlations using a two-tailed *t*-test (**p* < 0.05, ***p* < 0.01 and ****p* < 0.001). Rachis length (RL), spikelets number (SpN), primary branch number (PBN), secondary branch number (SBN), primary branch length (PBL), secondary branch length (SBL), PB internode length (PBintL), and SB internode length (SBintL), flowering time at early sowing (DFT2014a), flowering time at late sowing (DFT2014b), differential of flowering time (DIF2014).

**Supplementary Figure S8. Genome-wide association mapping of flowering time assessed for early sowing (DFTa)**. Manhattan plots were drawn using two years phenotypic data. (**A-B**) Manhattan plot (A) and Quantile-quantile (QQ) plot (B) drawn based on the latent linear mixed model (LFMM). (**C-D**) Manhattan plot (C)and QQ plot (D) drawn based on FarmCPU method. The dashed black lines represent the genome wide significance threshold (-log_10_ *p* = 4). Arrows correspond to the quantitative trait loci (QTLs) that co-localized DFTa.

**Supplementary Figure S9. Genomic organization and haplotype analysis for qRL3 (A)** Local Manhattan plots for qRL3. Red dots depict the region delimited to qRL3. The ideogram represents the genes that are expressed (black) and not expressed (grey) in the panicle based on the publicly available databases and RNA-seq data. Candidate genes known to be associated with panicle development are highlighted. (**B**) Heatmap showing linkage disequilibrium (LD) patterns for the chromosomal region around qRL3. Green vertical lines and black triangles indicate the SNP physical positions and the observed LD blocks for qRL3, respectively. LD is depicted by *r^2^* statistic. The light lime to red gradient depicts the range of *r^2^* values. (**C**) Haplotype network analysis of qRL3 performed using significant SNP markers. Haplotypes are denoted by circles with size corresponding to the number of accessions carrying that haplotype. (D-F) Boxplot with individual dots for rachis length (RL) based on two major haplotypes (n > 5 accessions), namely H1 and H2. The statistical difference between haplotypes were assessed by Welch’s *t*-test. (**G**) Lollipop plot showing the polymorphisms between haplotypes H1 and H2 in the qPBN3 region. The schematic view of the QTL (middle) depicts genes expressed in the panicle (black) and genes not expressed in the panicle (grey) according to the publicly available databases and RNA-seq data. The genes that are on the forward strand (positive-strand) are presented above x-axis and those that are on the reverse strand (negative-strand) are presented below x-axis. The different polymorphic sites between H1 and H2 haplotypes are represented by colored lollipops. To simplify the plot, only SNPs/InDels affecting protein sequences were represented (excluding synonymous changes and UTR/intronic sites) as well as only the InDels in the intergenic regions (full SNP/InDel list in supplementary Table S5).

**Supplementary Figure S10.** **Genomic organization and haplotype analysis for qSBN10.2. (A)** Local Manhattan plots for top. Blue dots and lines indicate the physical position of qSBN10.2. The ideogram represents the genes that are expressed (black) and not expressed (grey) in the panicle based on the publicly available databases and RNA-seq data. Candidate genes known to be associated with panicle development are highlighted. (**B**) Heatmap showing linkage disequilibrium (LD) patterns for the chromosomal region around the QTL. Green vertical lines and black triangles indicate the SNP physical positions and the observed LD blocks, respectively for qSBN10.2. LD is depicted by *r^2^* statistic. The light lime to red gradient depicts the range of *r^2^* values. (**C**) Haplotype network analysis of qSBN10.2 performed using significant SNP markers. Haplotypes are denoted by circles with size corresponding to the number of accessions carrying that haplotype. (D) Boxplot with individual dots for secondary branch number (SBN) based on the two major haplotypes (n > 5 accessions). The statistical difference between haplotypes were assessed by Welch’s *t*-test. (**G**) Lollipop plot showing the polymorphisms between haplotypes H1 and H2 in the qSBN10.2 region. The schematic view of the QTL (middle) depicts genes expressed in the panicle (black) and genes not expressed in the panicle (grey) according to the publicly available databases and RNA-seq data. The genes that are on the forward strand (positive-strand) are presented above x-axis and those that are on the reverse strand (negative-strand) are presented below x-axis. The different polymorphic sites between H1 and H2 are represented by colored lollipops. To simplify the plot, only SNPs/InDels affecting protein sequences were represented (excluding synonymous changes and UTR/intronic sites) as well as only the INDELs in the intergenic regions (full SNP/InDel list in supplementary Table S6).

**Supplementary Figure S11.** **Allelic diversity in *OgSET1* in the whole *O. glaberrima* panel.** (**A**) Haplotype analysis of *OgSET1* (Bottom) in 162 *O. glaberrima* genotypes using all the SNPs/InDels within the gene. The gene structure and polymorphic sites and their frequency in *O. glaberrima* population are shown on the top. The black box above the x-axis reveals that the targeted gene was expressed in the panicle based on the publicly available databases and RNA-seq data. The different polymorphic sites within the genes are represented by colored lollipops. The green, grey, and blue boxes represent UTRs, introns, and CDS regions of the *OgSET1* annotated transcript, respectively. (**B**) Boxplots with individual dots for primary branch number (PBN), spikelet number (SpN), secondary branch number (SBN), based on accessions from the 3 main haplotypes (n > 5 accessions) of *OgSET1* gene, namely Ha, Hb and Hc. The statistical difference between haplotypes were assessed by Welch’s *t*-test (NS: not significant; **p* < 0.05 and ***p* < 0.01).
