# Supplementary material for: Genome-wide association analysis identifies natural allelic variants associated with panicle architecture variation in African rice, Oryza glaberrima Steud
Source: G3 (Bethesda). 2023 Aug 3;13(10):jkad174. doi: 10.1093/g3journal/jkad174 (PMC10542218; doi:10.1093/g3journal/jkad174)

A

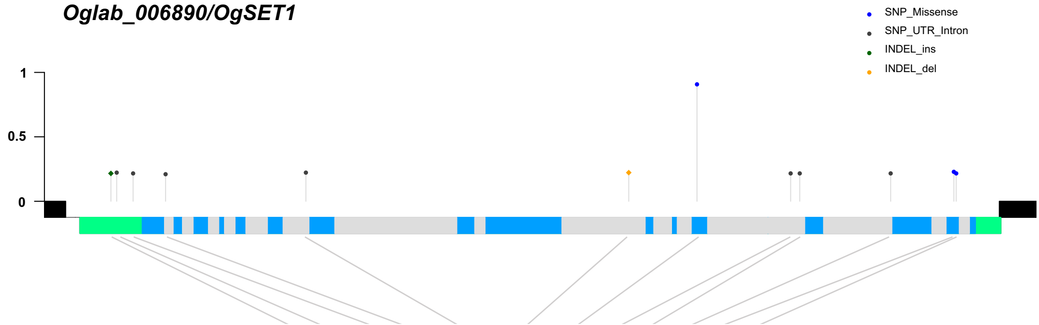

REF	G	C	T	T	G	GT	G	T	A	A	C	A	acc
ALT	GTT	T	A	C	A	G	C	C	T	C	T	T	nb
Hc	G	C	T	T	G	GT	G	T	A	A	C	A	15
Ha	G	C	T	T	G	GT	C	T	A	A	C	A	108
Hd	G	C	T	T	G	GT	C	T	A	A	T	A	1
He	G	C	T	T	G	G	C	T	A	A	C	A	1
Hf	G	C	T	T	A	GT	C	C	T	C	T	T	1
Hg	G	T	T	T	G	GT	C	T	A	A	T	A	1
Hh	GTT	T	A	T	A	G	C	T	A	A	C	A	1
Hb	GTT	T	A	C	A	G	C	C	T	C	T	T	34
Pos	10393			10394	10395	10397	10398	10399		10400			
	578	627	766	41	229	963	541	334	441	181	716	736	

B

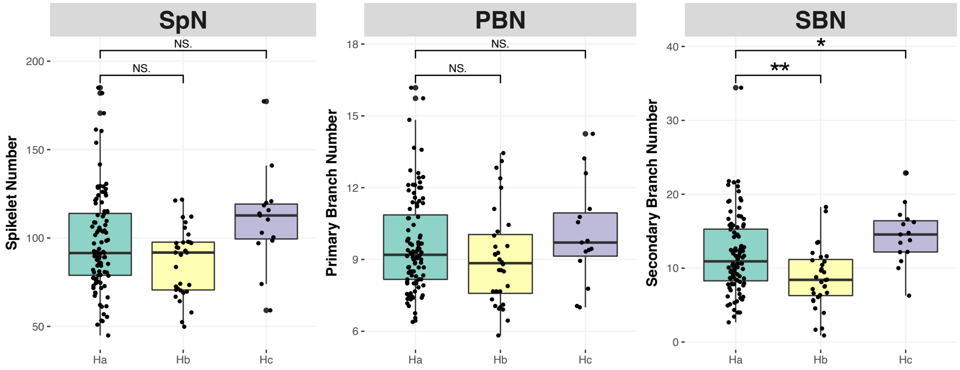

Supplement: jkad174_Supplementary_Data [file jkad174_supplementary_data.zip › Supplementary_Figure_S11_G3-2023-404399.pdf]
