# Supplementary figures and images for: Genome-wide association analysis identifies natural allelic variants associated with panicle architecture variation in African rice, Oryza glaberrima Steud
Source: G3 (Bethesda). 2023 Aug 3;13(10):jkad174. doi: 10.1093/g3journal/jkad174 (PMC10542218; doi:10.1093/g3journal/jkad174)

**A**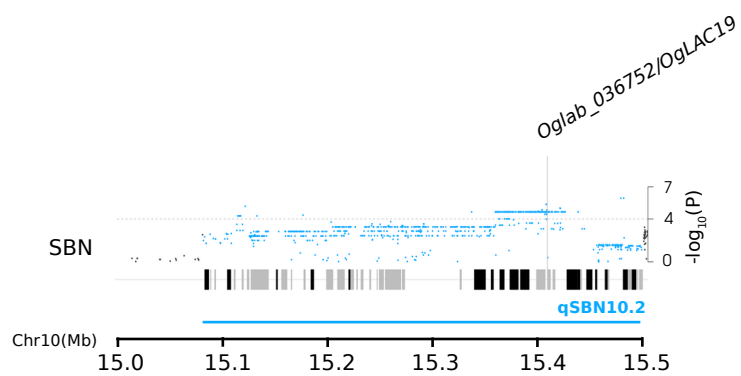**C**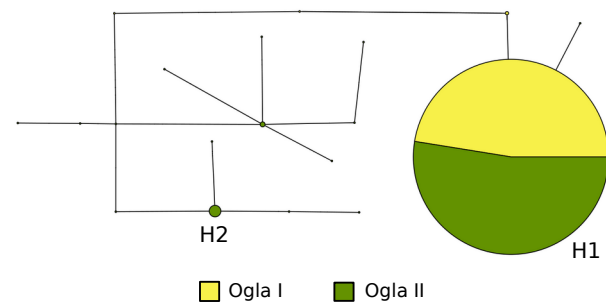**B**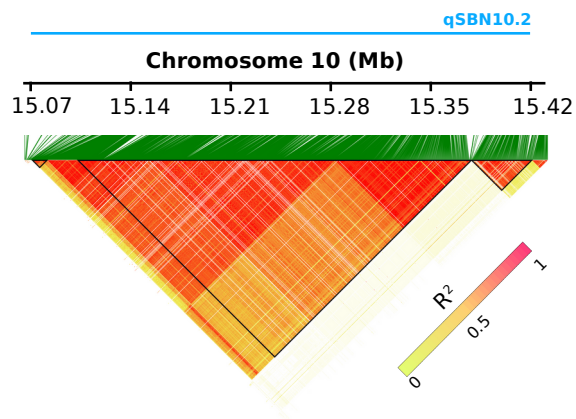**D**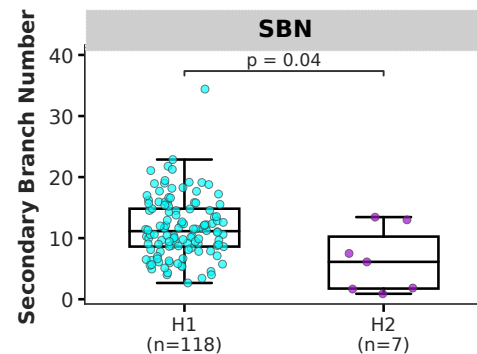**E**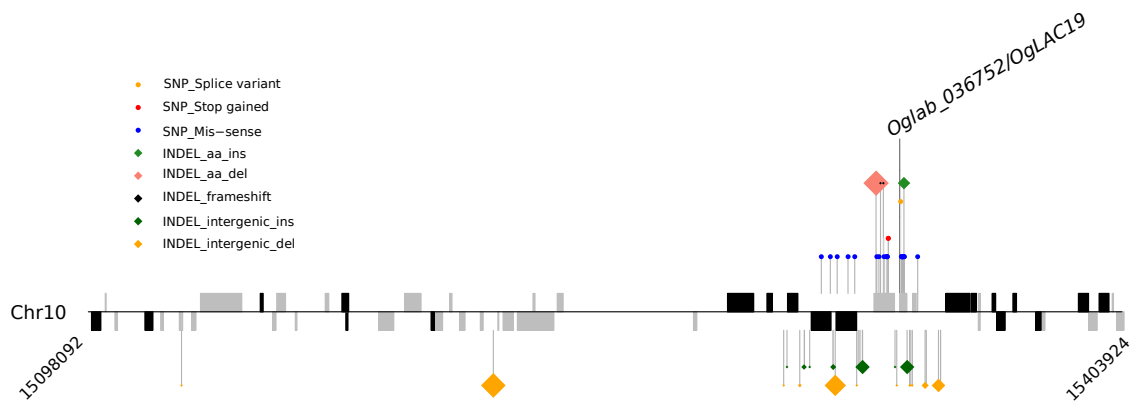

Supplement: jkad174_Supplementary_Data [file jkad174_supplementary_data.zip › Supplementary_Figure_S10_G3-2023-404399.pdf]

A

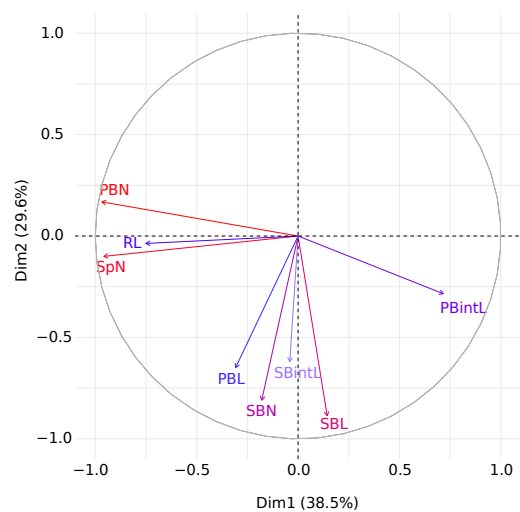

2012

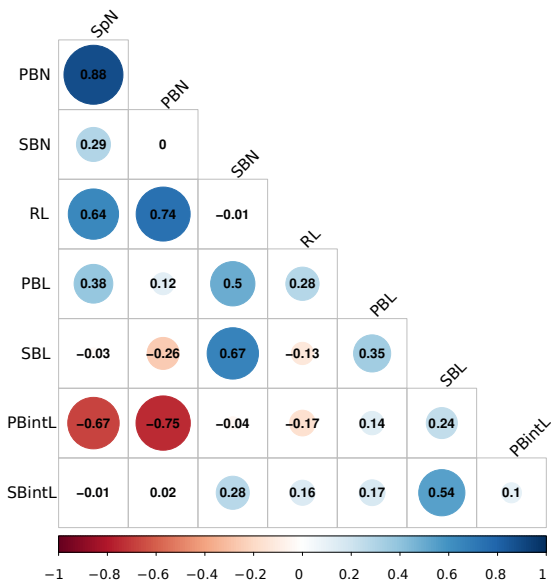

B

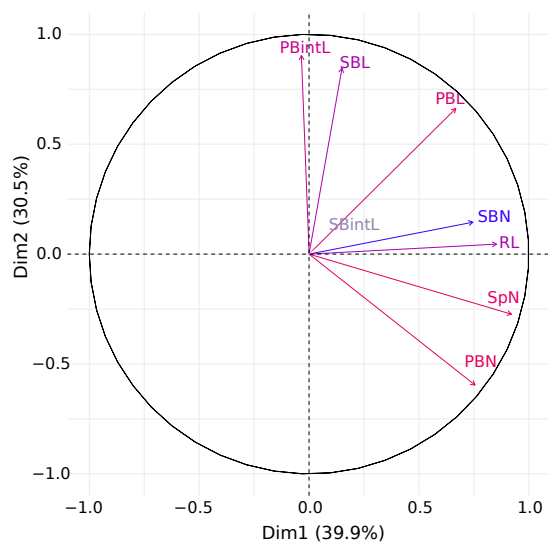

2014

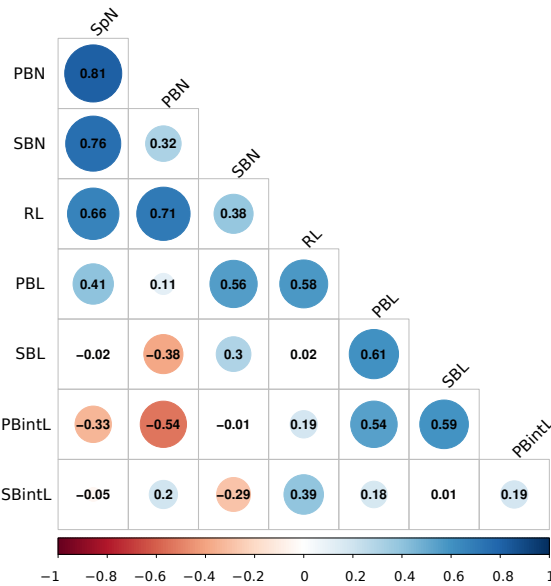

C

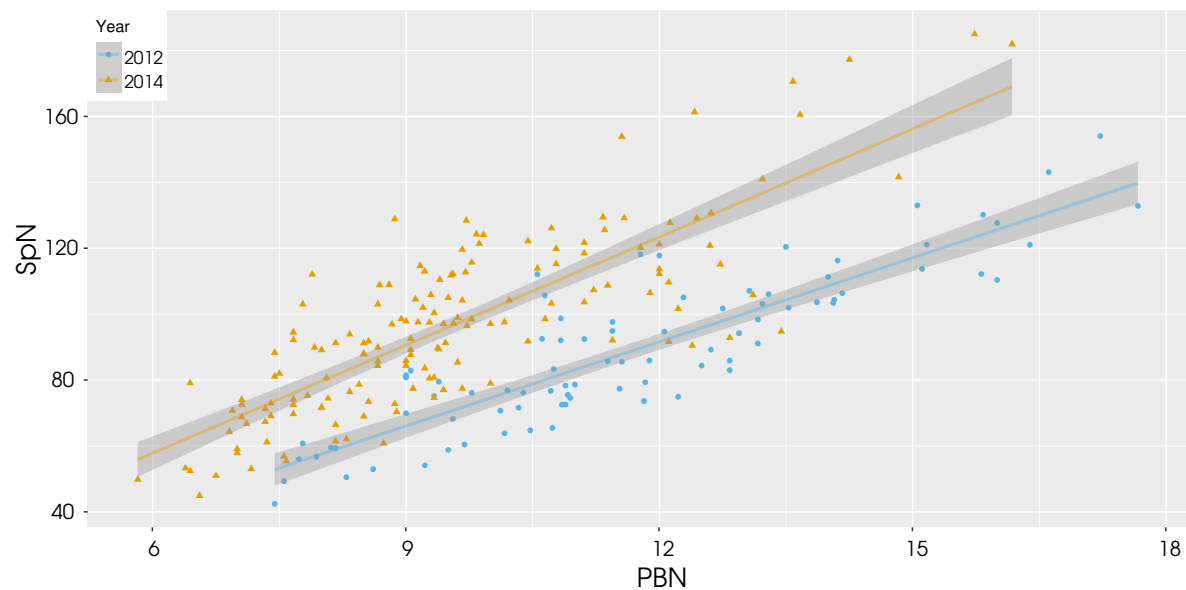

Supplement: jkad174_Supplementary_Data [file jkad174_supplementary_data.zip › Supplementary_Figure_S1_G3-2023-404399.pdf]

## Overall LD decay

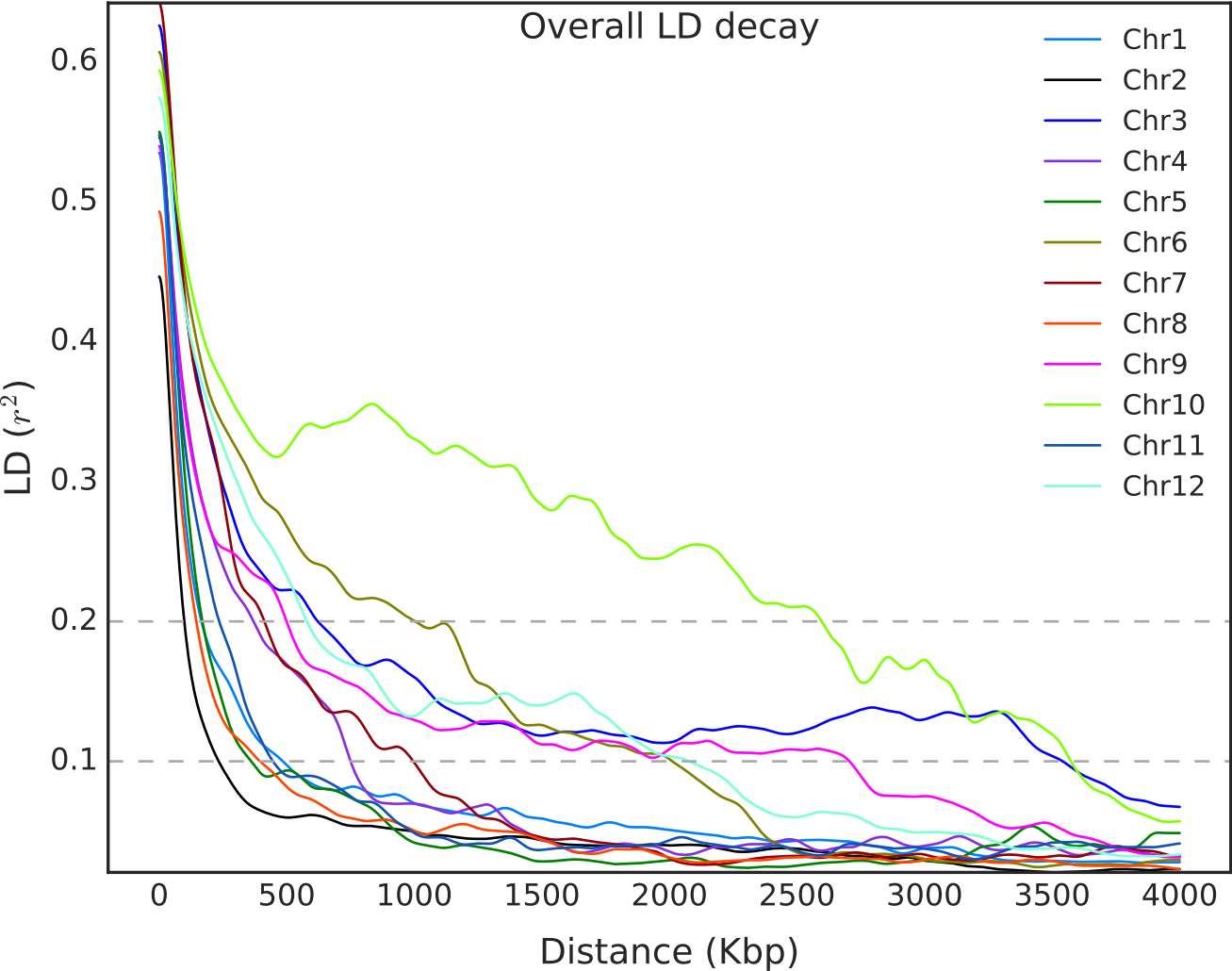

Supplement: jkad174_Supplementary_Data [file jkad174_supplementary_data.zip › Supplementary_Figure_S2_G3-2023-404399.pdf]

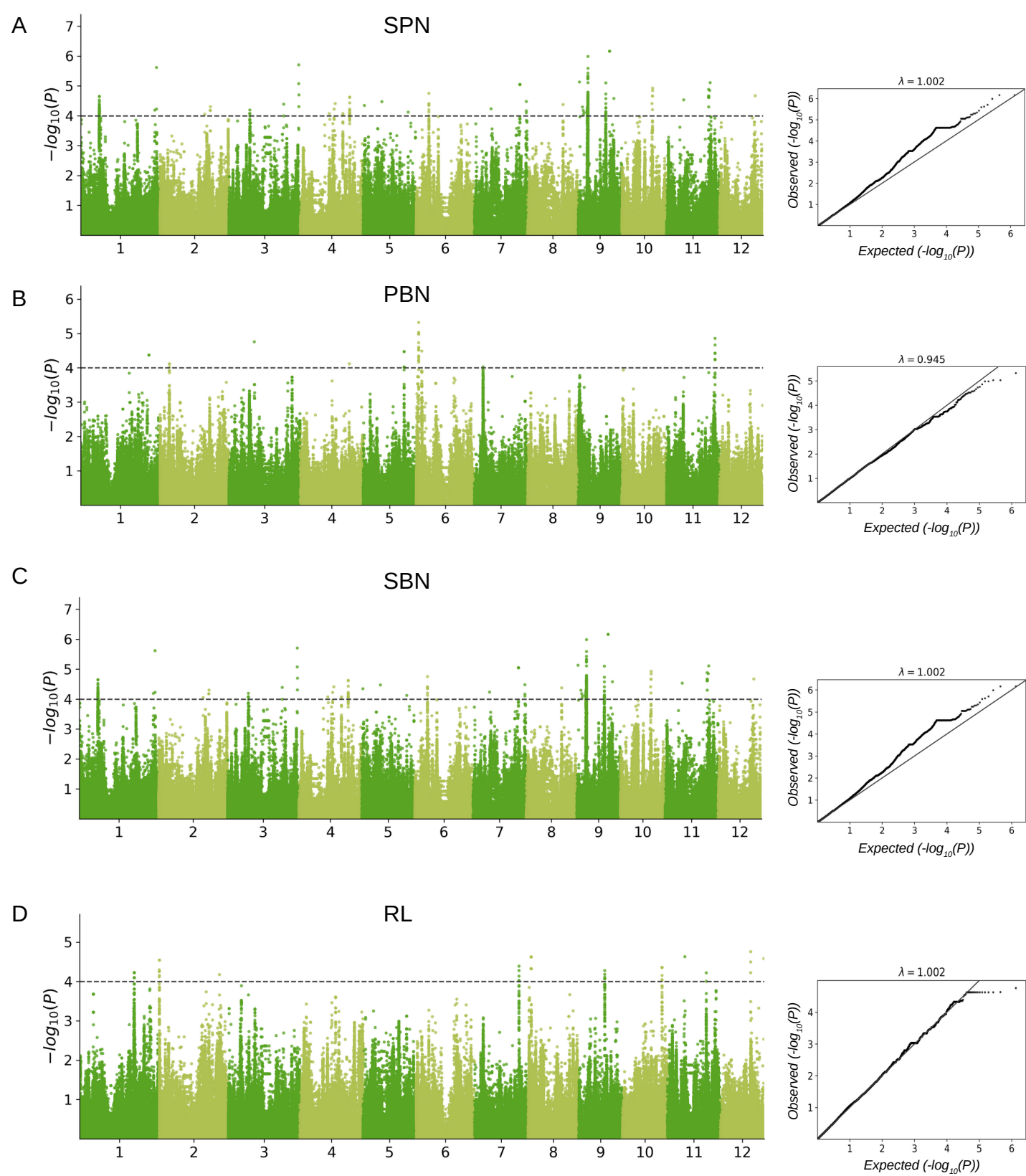

Supplement: jkad174_Supplementary_Data [file jkad174_supplementary_data.zip › Supplementary_Figure_S3_G3-2023-404399.pdf]

A

SPN

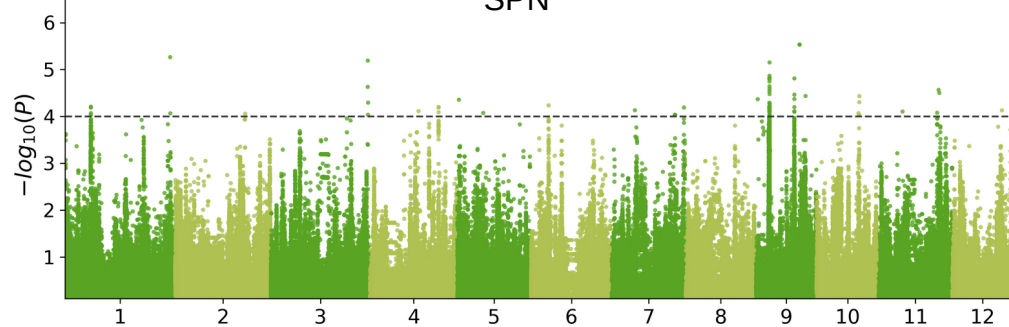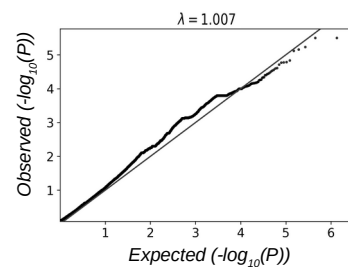

B

PBN

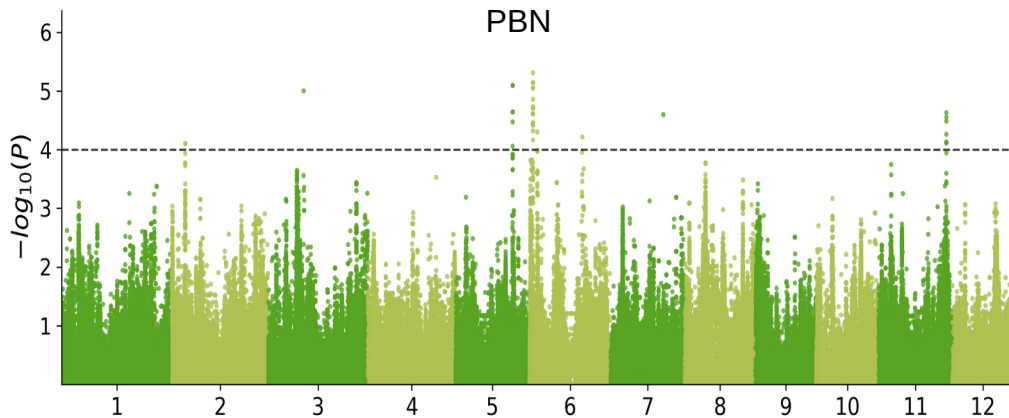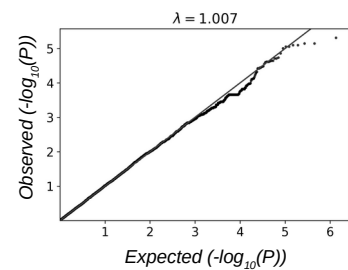

C

SBN

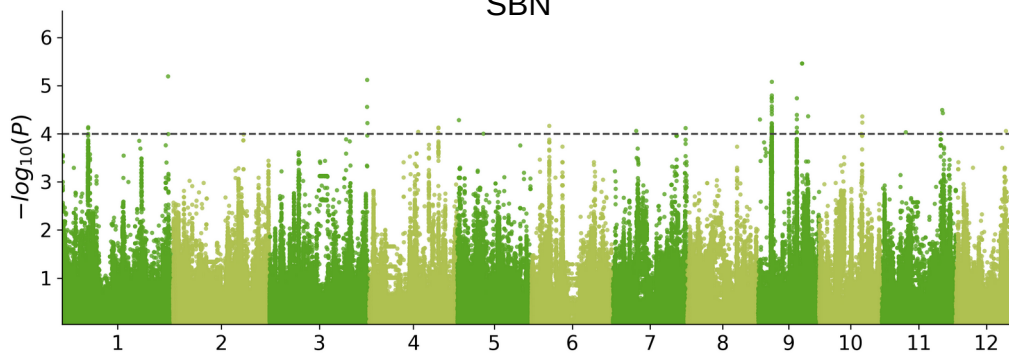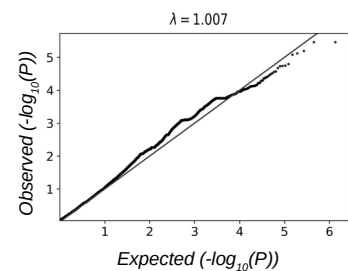

D

RL

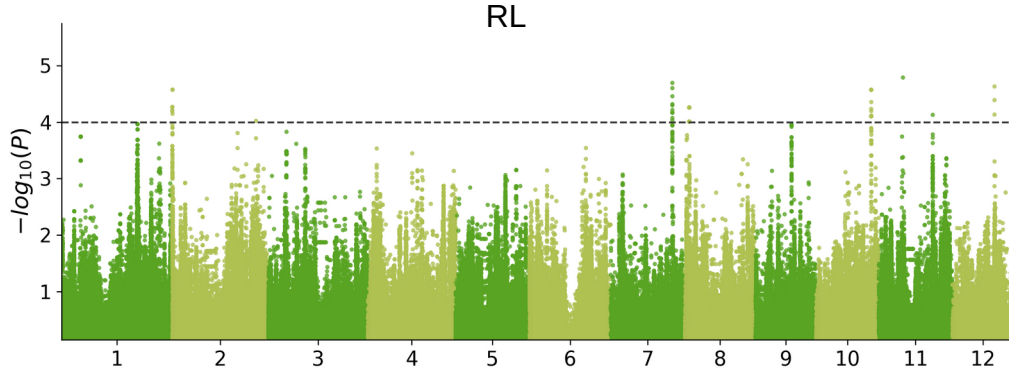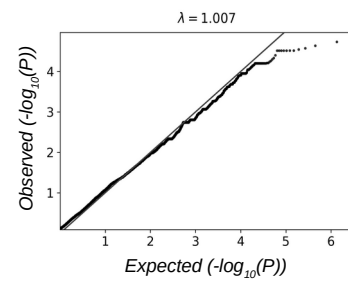

Supplement: jkad174_Supplementary_Data [file jkad174_supplementary_data.zip › Supplementary_Figure_S4_G3-2023-404399.pdf]

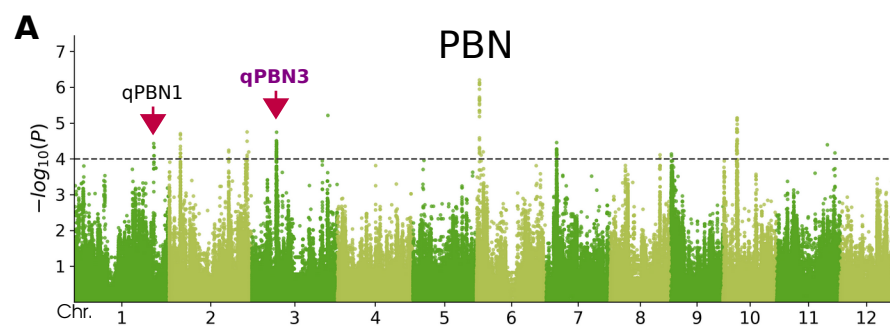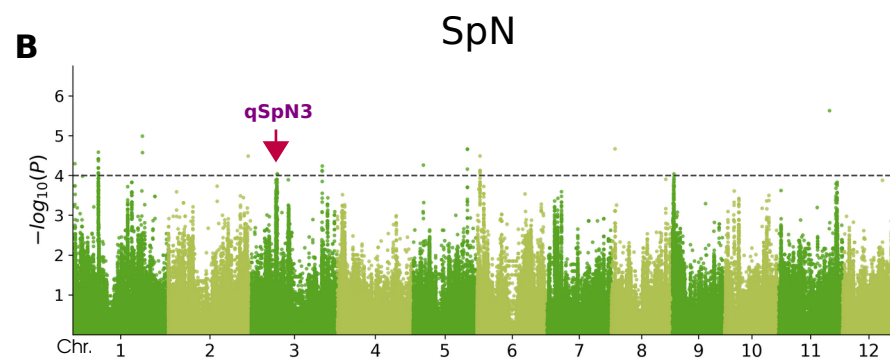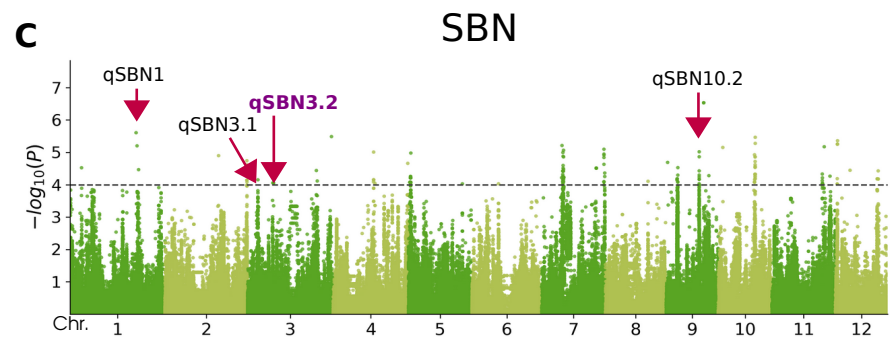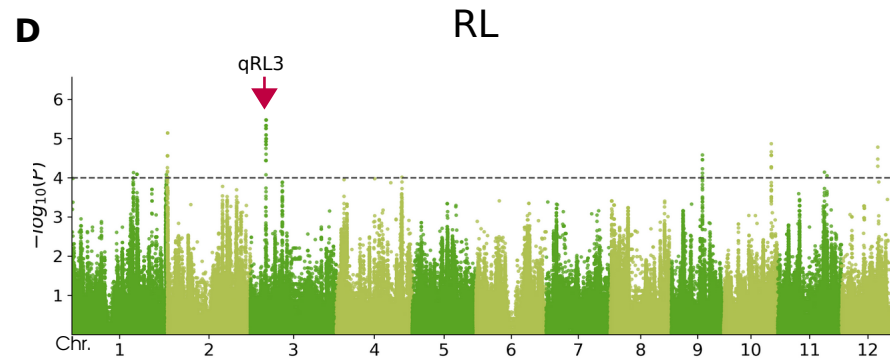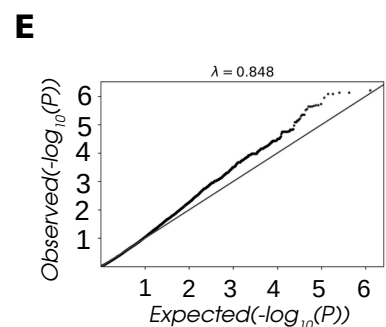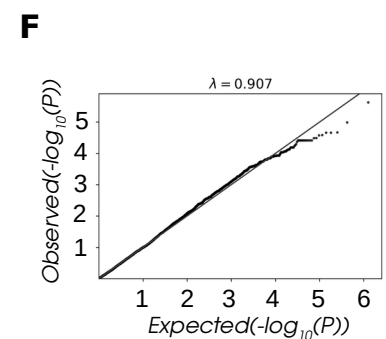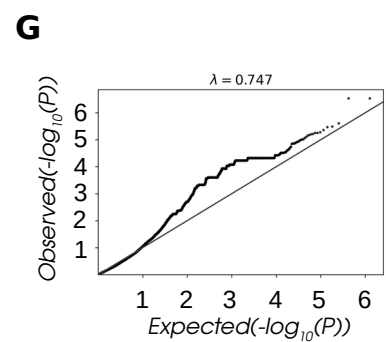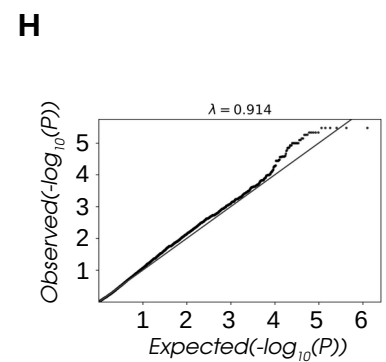

Supplement: jkad174_Supplementary_Data [file jkad174_supplementary_data.zip › Supplementary_Figure_S5_G3-2023-404399.pdf]

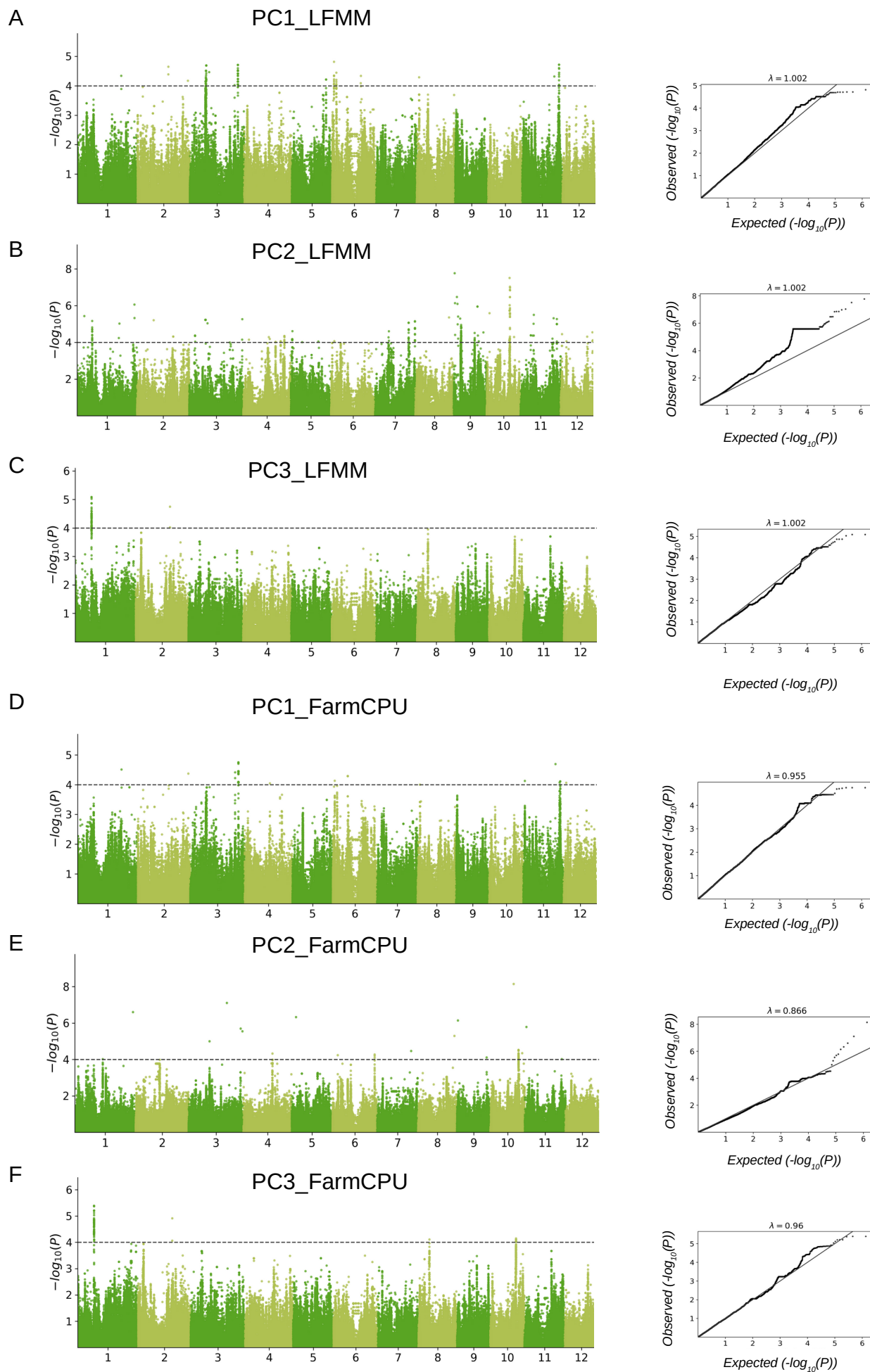

Supplement: jkad174_Supplementary_Data [file jkad174_supplementary_data.zip › Supplementary_Figure_S6_G3-2023-404399.pdf]

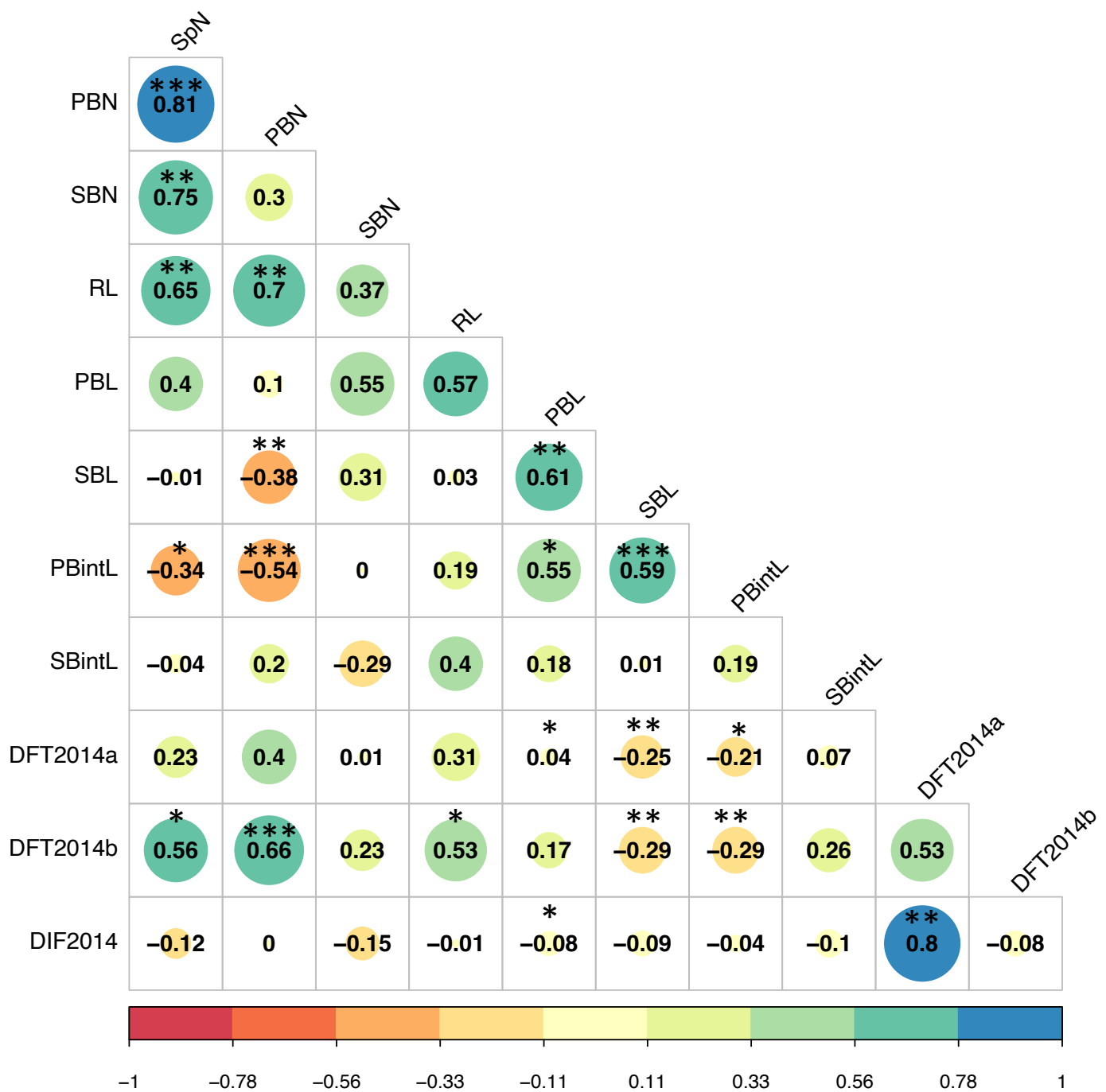

Supplement: jkad174_Supplementary_Data [file jkad174_supplementary_data.zip › Supplementary_Figure_S7_G3-2023-404399.pdf]

# DFTa\_LFMM

**A**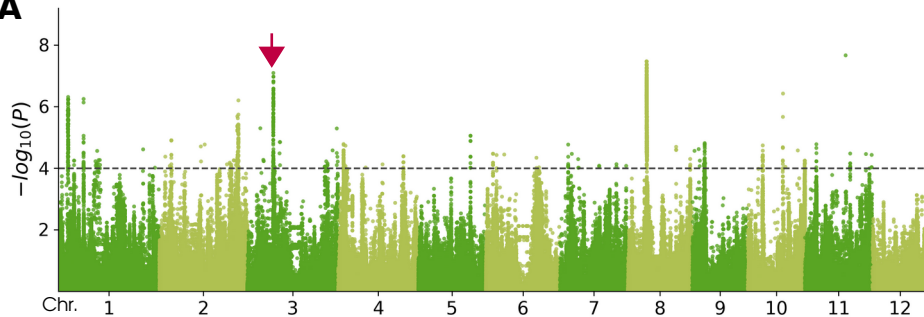**C**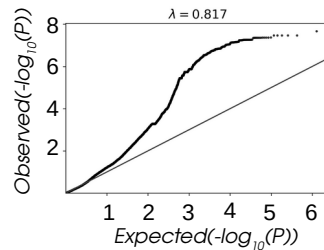**B**

# DFTa\_FarmCPU

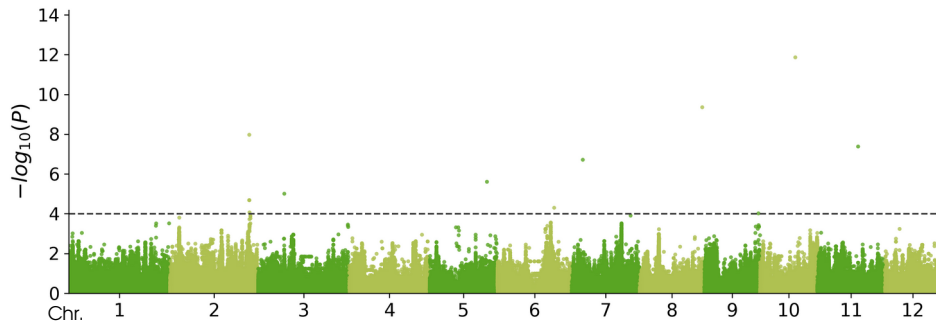**D**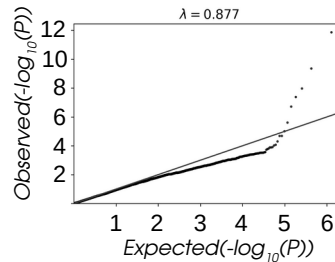

Supplement: jkad174_Supplementary_Data [file jkad174_supplementary_data.zip › Supplementary_Figure_S8_G3-2023-404399.pdf]

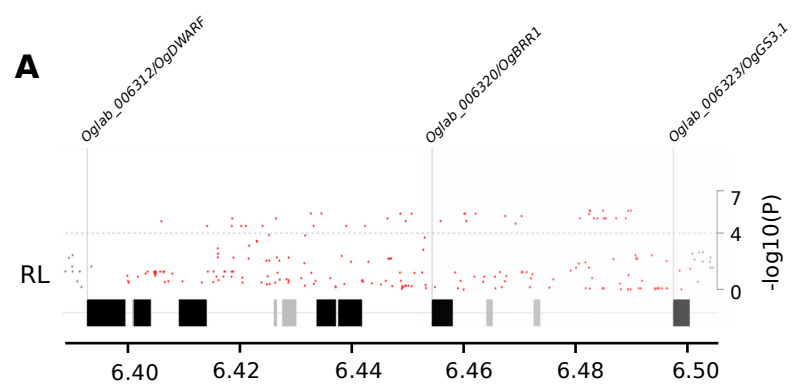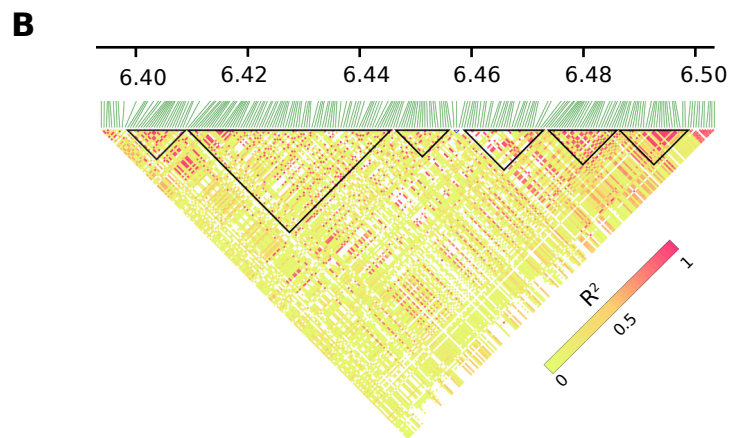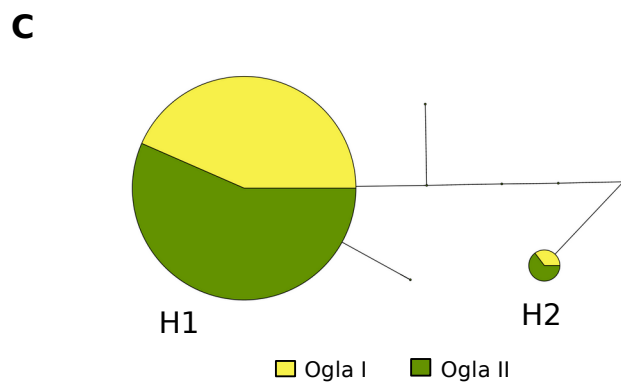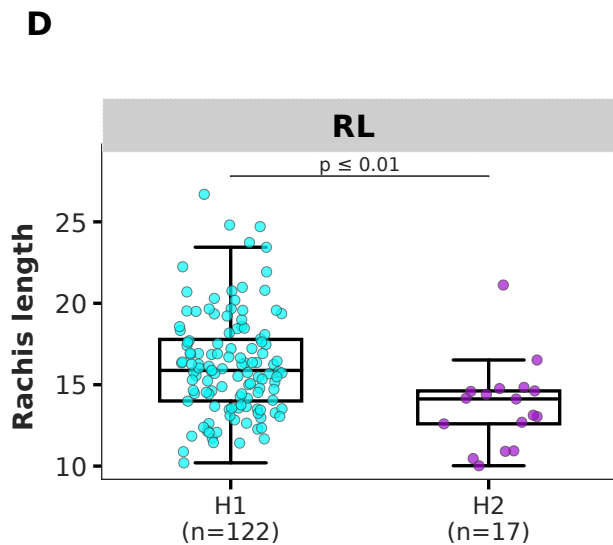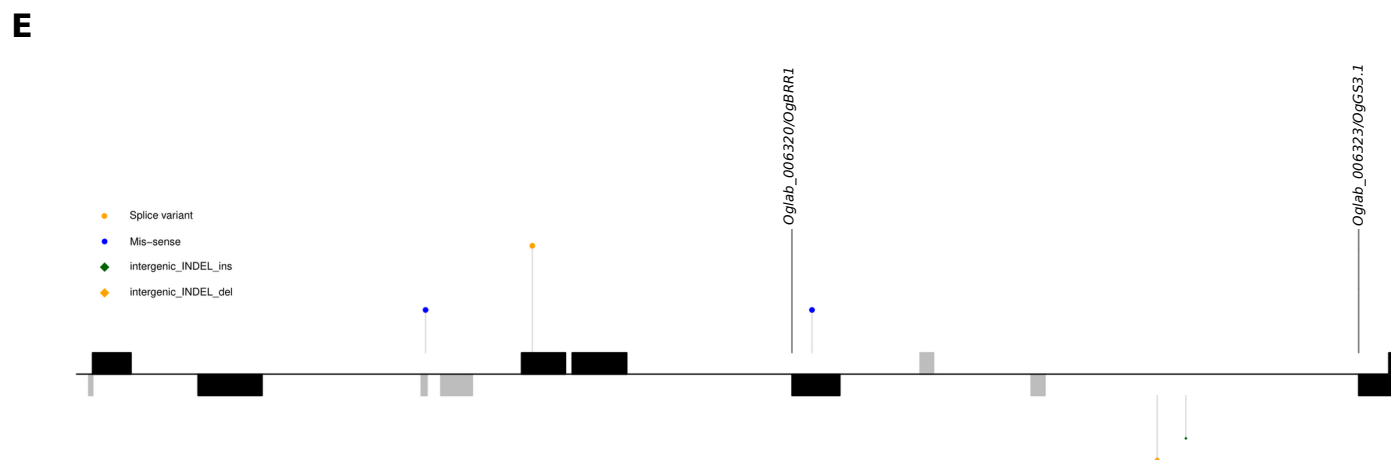

Supplement: jkad174_Supplementary_Data [file jkad174_supplementary_data.zip › Supplementary_Figure_S9_G3-2023-404399.pdf]
